# *Dictyocaulus viviparus* genome, variome and transcriptome elucidate lungworm biology and support future intervention

**DOI:** 10.1038/srep20316

**Published:** 2016-02-09

**Authors:** Samantha N. McNulty, Christina Strübe, Bruce A. Rosa, John C. Martin, Rahul Tyagi, Young-Jun Choi, Qi Wang, Kymberlie Hallsworth Pepin, Xu Zhang, Philip Ozersky, Richard K. Wilson, Paul W. Sternberg, Robin B. Gasser, Makedonka Mitreva

**Affiliations:** 1 The McDonnell Genome Institute, Washington University in St Louis, MO 63108, USA; 2 Institute for Parasitology, University of Veterinary Medicine Hannover, Hannover 30559, Germany; 3 HHMI, Division of Biology, California Institute of Technology, Pasadena, CA 91125, USA; 4 Faculty of Veterinary and Agricultural Sciences, The University of Melbourne, Victoria 3010, Australia; 5 Division of Infectious Diseases, Department of Medicine, Washington University School of Medicine, St. Louis, MO 63110, USA

## Abstract

The bovine lungworm, *Dictyocaulus viviparus* (order Strongylida), is an important parasite of livestock that causes substantial economic and production losses worldwide. Here we report the draft genome, variome, and developmental transcriptome of *D. viviparus*. The genome (161 Mb) is smaller than those of related bursate nematodes and encodes fewer proteins (14,171 total). In the first genome-wide assessment of genomic variation in any parasitic nematode, we found a high degree of sequence variability in proteins predicted to be involved host-parasite interactions. Next, we used extensive RNA sequence data to track gene transcription across the life cycle of *D. viviparus*, and identified genes that might be important in nematode development and parasitism. Finally, we predicted genes that could be vital in host-parasite interactions, genes that could serve as drug targets, and putative RNAi effectors with a view to developing functional genomic tools. This extensive, well-curated dataset should provide a basis for developing new anthelmintics, vaccines, and improved diagnostic tests and serve as a platform for future investigations of drug resistance and epidemiology of the bovine lungworm and related nematodes.

Parasitic roundworms (nematodes) of domestic animals are responsible for substantial economic losses as a consequence of poor production performance, morbidity, and mortality^1^. Diseases caused by these worms cost livestock industries billions of dollars and lead to a significant reduction in global food production each year^2^. Lungworms of the genus *Dictyocaulus* (Strongylida: Dictyocaulidae, nematode clade V) are parasites of major agricultural significance that cause parasitic bronchitis (or dictyocaulosis) in cattle, particularly in young animals^3^. The clinical manifestation of dictyocaulosis vary from mild respiratory signs to emphysema and pneumonia, and can result in rapid death in severely affected animals^1^,^3^. Respiratory symptoms are accompanied by a reduction in growth, fertility and/or milk production^3^ such that outbreaks result in major financial losses to farmers^4^.

Unlike other trichostrongylid nematodes that live in the upper alimentary tract, *D. viviparus* resides in the bronchi and bronchioles of the lungs (Fig. 1). Ovo-viviparous females release eggs that are coughed up and swallowed. The eggs hatch as they pass through the gastrointestinal tract, and first stage larvae (L1) are shed in the faeces. Larvae moult twice on the pasture to develop into infective third-stage larvae (L3s). This development can occur within four to six days under favorable environmental conditions. During this time, they must move from faeces to grass to be accessible to a host, and often take advantage of sporulating *Pilobilus* fungi for dispersal^5^. Upon ingestion by the bovid, L3s exsheath, penetrate the intestinal wall, migrate to the mesenteric lymph nodes and moult to the fourth larval stage (L4s). L4s are carried to the lung in circulating blood and/or lymph, and undergo a final moult to reach the L5, pre-adult stage (also referred to as immature adults). Although this is the last moult, further growth and development are required to reach sexual maturity. Most adult worms will be cleared by 30 days post-infection^6^; however, larval stages can undergo arrested development (hypobiosis) in the host lungs for up to five months if they were exposed to cold conditions prior to ingestion^7^. Hypobiosis is a crucial aspect of lungworm epidemiology and acts as a key factor for year-to-year survival in temperate climates^8^.

Farmers need to take routine measures to protect their livestock from lungworm infection due to the severe impact of dictyocaulosis on bovine health. An irradiated larval vaccine was introduced in the 1950‘s; it is highly effective, but has a short shelf-life and, more importantly, booster infections or vaccination are necessary to confer immunity beyond six to 12 months^9^. Recombinant vaccines have been attempted, but failed to achieve adequate levels of protection^10^,^11^. *D. viviparus* is susceptible to several classes of drugs, including macrocyclic lactones and benzimidazoles^3^; thus, anthelmintic drugs have superseded vaccination as the preferred preventative method. However, prophylactic treatment of calves impedes the development of protective immunity that would shield them from disease later in life^3^. In addition, excessive and widespread drug use promotes resistance in nematode populations, and there are reports of resistance developing in *D. viviparus*^12^,^13^. The discovery of new interventions has been challenging due to a limited understanding of the biology this parasite.

In the present study, we characterize the draft genome, population variome and developmentally-staged transcriptome of *D. viviparus* in order to substantially improve our understanding of this pathogen at the molecular level across all defined life cycle stages and its relationship with the bovine host. The Hannover Dv2000 field isolate, a temperate strain from Northern Germany, was selected for this investigation because it has been the subject of previous recombinant vaccine^14^ and gene expression studies^15^,^16^,^17^, serves as the basis for the lungworm-MSP-ELISA diagnostic assay^18^,^19^, and (unlike the strain used for producing live vaccine) is capable of undergoing hypobiosis. This is the first comprehensive “omic” study of an economically important nematode parasite of the respiratory system.

## Results and Discussion

### Genome features

The nuclear genome of *D. viviparus* Hannover Dv2000 was sequenced and assembled into 7,157 contigs (N50: 225,748 bp) with a total length of 161 Mb and GC content of 34.8% (Table 1, Supplementary Table S1). Completeness was estimated at 99% based on presence/absence of essential eukaryotic genes. Compared with related nematodes, the *D. viviparus* genome is considerably smaller than the gastrointestinal parasites *Haemonchus contortus* (320–370 Mb)^20^,^21^ and *Necator americanus* (244 Mb)^22^ but larger than that of the free-living worm *Caenorhabditis elegans* (100 Mb, WormBase WS230). The repeat content of the *D. viviparus* genome was estimated at 17.5% (Supplementary Table S2), less than *H. contortus* (29.0%) and *N. americanus* (23.5%), which might contribute to its smaller genome size.

A total of 14,171 protein-coding genes were predicted from the *D. viviparus* genome (Table 1, Supplementary Table S3), more than 99% of which were supported by RNA-seq data in our developmental transcriptome dataset. In contrast, other strongylid nematodes have gene counts in the order of 20,000^20^,^21^,^22^. The average length of *D. viviparus* protein coding sequences (983 bp, including only exons) was somewhat larger than the average lengths reported for *H. contortus* and *N. americanus*; however, the average gene footprint size was much smaller (3,080 bp for *D. viviparus* compared with 4,289 bp for *N. americanus* and 6,167 or 6,564 bp for *H. contortus*; see Supplementary Table 1), as there appear to be fewer and shorter introns. The reduced gene count and gene footprints may also contribute to the smaller overall genome size.

### Gene order and organization

We matched the protein coding genes of *D. viviparus* to their orthologs in *C. elegans* to examine synteny and colinearity between the two species (Supplementary Table S3). *D. viviparus* genes on the same supercontig usually had orthologs on a particular *C. elegans* chromosome (Fig. 2a). For example, the contig D_viviparus-1.0_Cont1 encodes 207 genes; 112 of them have orthologs in *C. elegans*, 94 of which are on chromosome V. Despite the preservation of physical linkage, gene order was not well conserved. Short regions of co-linearity with *C. elegans* (at least five genes in order) occur on just seven scaffolds, one of which is depicted in Fig. 2b. This finding is consistent with comparisons between *C. elegans* and other parasitic nematodes such as *H. contortus* and *Trichinella spiralis*^20^,^23^.

Approximately 17% of all *C. elegans* genes are organized into operons (groups of two or more contiguous genes regulated by a single promoter and transcribed as a single RNA molecule). The resultant polycistronic mRNAs are cleaved into single-gene units (cistrons), which are trans-spliced to spliced leader (SL) sequences. Usually, a well-conserved SL1 sequence is attached to the 5′-end of the first cistron in the operon, and a less-conserved SL2 sequence is attached to the 5′-end of subsequent cistrons (though SL1s are also known to be attached to subsequent cistrons). In total, 363 operons containing 793 genes were identified in *D. viviparus* based on orthology to known *C. elegans* operons (Supplementary Table S4); 188 operons had a conserved order and orientation, whereas 175 were partly conserved, with missing genes or an altered order or orientation (Fig. 2c). As expected, RNA-seq data confirmed the co-transcription of operon genes as compared non-operon genes, even accounting for gene proximity (P < 2 × 10^−11^; Supplementary Fig. S1). A total of 4,633 (32.7%) *D. viviparus* genes were associated with an SL1 sequence (Supplementary Table S3); only a small subset of them (n = 370) belonged to the operons identified based on orthology to *C. elegans*. We were only able to find 77 SL2-associated genes due to the limited conservation of SL2 sequences (Supplementary Table S3); only nine of these appear to be part of operons based on their proximity (<4,000 nt) to other SL2 genes.

### Annotation of the deduced proteins

Putative functions were assigned to the 14,171 *D. viviparus* proteins based on similarity to sequences in publicly available databases (Supplementary Table S3; Supplementary Methods). A total of 4,769 unique InterPro protein domains were predicted from 9,198 (64.9%) *D. viviparus* proteins, which were associated with 1,388 unique gene ontology (GO) terms. Likewise, 8,442 (59.6%) proteins matched 4,102 unique KEGG orthologous groups, which were further assigned to 324 enzymatic pathways and 242 pathway modules. The majority (91.0%) of *D. viviparus* proteins had at least one BLAST match (e-value < 10^−5^) in the NCBI non-redundant protein database (nr), with most corresponding to proteins from other strongylid nematodes.

BLAST searches against nr identified 55 *D. viviparus* proteins with isolated regions of similarity to sequences from *Wolbachia* spp. (Supplementary Table S3). *Wolbachia* are obligate bacterial endosymbionts of some arthropods and filarial nematodes and are implicated in the transfer of DNA to host nuclear genomes. While *D. viviparus* does not presently host *Wolbachia* endobacteria, the bacterial sequences in its nuclear genome suggest that it (or an ancestral species) was infected in the past. These inserts have been thoroughly described in a report based on the genome of a *D. viviparus* strain from Cameroon^24^. The focus of this report was the *Wolbachia*-like sequence inserts, not the *D. viviparus* genome itself, so the genome and gene complement of the nematode were only briefly described and little functional annotation was provided (see Supplementary Table S1 for a brief comparison of the two assemblies). Although the previous report claimed little evidence of transcriptional of *Wolbachia* sequences^24^, 53 of the 55 protein coding genes with *Wolbachia*-like sequences showed evidence of transcription based on our extensive RNA-seq data. However, they were transcribed at low levels compared with other genes (average peak expression of 77.7 FPKM compared with 3,356.6 FPKM for other genes; P < 10^−10^, t-test using log-scale FPKM values; Supplementary Fig. S2), and did not exhibit any distinct expression pattern across the parasite life cycle (Supplementary Table 3). A majority of the *Wolbachia*-like genes identified in other *Wolbachia*-free species show limited transcription and no detectable protein expression^25^. Thus, findings were consistent with these expectations.

### Phylogenetic conservation of the deduced proteins

To further assess the conservation of proteins among parasitic nematodes of the phylum Nematoda (representing strongylids, trichinellids, and filarioids/spiruroids), their hosts, and outgroups, proteins from 16 species were clustered into orthologous protein families (OPFs; Supplementary Table S3). The “birth” and “death” of OPFs throughout evolution (Fig. 3a) was consistent with previously described patterns^23^,^26^, where OPF births outnumbered deaths in the major lineages such as Nematoda. Divergence is observed for the majority of the terminal lineages, as reflected in a loss of OPFs. *D. viviparus* shows a greater number of OPF deaths than other nematodes, consistent with its reduced geneset. Some 835 OPFs contained genes from *H. contortus, N. americanus*, and *C. elegans* but not *D. viviparus*; these included 916 genes from *N. americanus* that were significantly enriched for GO terms related to steroid hormone-mediated signaling (Supplementary Table S5).

In total, 401 OPFs were specific to *D. viviparus* among clade V nematodes (Fig. 3b), and 121 OPFs (representing 365 proteins) were specific to *D. viviparus* among all 16 species included in this analysis. A further 3,194 *D. viviparus* proteins were classified as singletons (no orthologs or paralogs; not included in OPFs). Of the 3,559 total proteins considered *D. viviparus*-specific in the OPF analysis, 1,281 had no significant hits to proteins in nr. The only significantly enriched InterPro protein domain among the 3,559 *D. viviparus*-specific proteins was the CAP (cysteine-rich secretory proteins, antigen 5, and pathogenesis-related 1 proteins) domain (Supplementary Table S6, IPR014044; *P* = 9 × 10^−6^), the representative domain for SCP/TAPS (sperm-coating protein/Tpx/antigen 5/pathogenesis related-1/Sc7) proteins^27^. CAP domain proteins are involved in nematode-host interactions (described below). Besides the 121 *D. viviparus* specific OPFs, six other OPFs (containing 95 *D. viviparus* genes) were significantly expanded (*P < *10^−10^) in *D. viviparus* compared with the other nematodes studied (Supplementary Table S3), including OG_IF1.51110, which contained 41 chymotrypsin orthologs in *D. viviparus* compared with only six in *N. americanus* and one in each *T. spiralis* and *C. elegans.* Serine peptidases, including chymotrypsin, have been implicated as important factors for facilitating disease in other parasitic nematodes such as *N. americanus*^22^, *Trichuris muris*^28^, and *Ancylostoma ceylanicum*^29^. Two more expanded OPFs included serpentine GPCRs (OG_IF1.53882, 13 genes, *P* = 2 × 10^−10^; OG_IF1.55659, 7 genes, *P* = 0.04), which have been suggested as drug targets due to their key role(s) in nematode chemoreception^22^,^30^. These expanded OPFs warrant further exploration to better understand *D. viviparus* speciation.

### Population genomics of *D. viviparus*

Studying genetic variation is central to understanding the population structures, evolution and epidemiology of parasites. Previous studies have explored genetic variation in *D. viviparus*^31^,^32^,^33^, but were restricted to the mitochondrial genome or polymorphism in selectively amplified regions. Here, we sequenced nine individual adult worms (four males and five females) from the same host in order to estimate genetic diversity within an intra-host population. Sequence data from the previously reported Cameroon strain were also included in our analysis to assess differences between geographically distinct isolates. Mapping reads to our reference genome (Hannover Dv2000) resulted in an average 98% breadth and 58.1x depth of mapped read coverage with 89.1% informative sites (i.e., sites with sufficient mapping depth to facilitate variant calling) across the ten samples. Following variant calling and quality filtering, we identified 3,694,482 high-confidence single nucleotide polymorphisms (SNPs). Overall, the average genetic distance was less among the females as compared with males for the Hannover population (*π*_female_ = 0.0070; *π*_male_ = 0.0084; *π*_total_ = 0.0074), and there was little partitioning of the genetic diversity between the genders (Weir and Cockerham mean *F*_*ST*_ estimate = 0.0006; weighted *F*_*ST*_ estimate = 0.0085), consistent with the nested population structure depicted in the MDS plot (Fig. 4a). A haplotype network analysis of the mitochondrial genome of the ten worms provided a complementary view of the population, suggestive of four distinct maternal haplotypes in the Hannover worms (Fig. 4b).

Tajima’s D and the ratio of nonsynonymous to synonymous polymorphism rates (π_N_/π_S_) were calculated for each gene to identify genes that appear to be under selection (Fig. 4c). Negative Tajima’s D is a potential indication of purifying selection. The 5% of genes with the lowest Tajima’s D value are significantly more likely than other genes to be conserved across all species in the orthology analysis (P = 2 × 10^−6^) (Supplementary Table 3); reduced sequence diversity in the present population seems consistent with conservation throughout the phylogenetic lineage. In contrast, positive Tajima’s D could be indicative of either (1) balancing selection or (2) a partial selective sweep. We used π_N_/π_S_ to help distinguish between these two possibilities, as π_N_ and π_S_ are expected to increase proportionally prior to fixation of a favorable allele. Some 16 genes were found to have high Tajima’s D (top 5%) and positive log(π_N_/π_S_) values, suggestive of balancing selection. The 5% of genes with the highest π_N_/π_S_ (regardless of Tajima’s D) were enriched for being *D. viviparus*-specific in the orthology analysis (P < 10^−10^). Interestingly, these were also enriched for *Wolbachia*-like sequences (P = 0.02) and for genes with secretion signals (P = 0.007).

We explored non-synonymous SNP rates independently in order to assess primary protein sequence diversity in the intra-host population. While this is not necessarily indicative of selection, high levels of protein sequence diversity could have practical implication for drug or diagnostic target selection. Altogether, 5,308 genes were associated with one or more non-synonymous SNPs in the population. The 708 genes with the top 5% highest non-synonymous SNP rates (per amino acid; Supplementary Table S3) among the Hannover samples were significantly more likely than other genes to contain secretion signals (*P* = 6 × 10^−4^) and to be *D. viviparus*-specific in the OPF analysis (*P*  <10^−10^). These genes were also enriched for several InterPro domains, including the CAP domain (IPR014044; *P*  <10^−10^) and three domains related to peptidase activity (IPR001254, IPR009003 and IPR018114), which were similarly supported by multiple GO term enrichments (Supplementary Tables S6 and S7).

### Gene transcription throughout development

Next, we explored gene expression throughout the life cycle of *D. viviparus*. A total of 11,179 genes (79%) were transcribed, to some degree, in every sampled life cycle stage; 1,102 of these are considered constitutively expressed given that they showed no statistically significant variation over the course of the life cycle. Conversely, 12,681 (90%) were differentially expressed across the development of the parasite (Supplementary Fig. S3, Supplementary Table S8). Clustering based on transcription patterns showed that major points of differentiation include the transitions from egg to L1, from free-living to parasitic stages, and between female and male adults (Fig. 5a).

**Figure 1:**
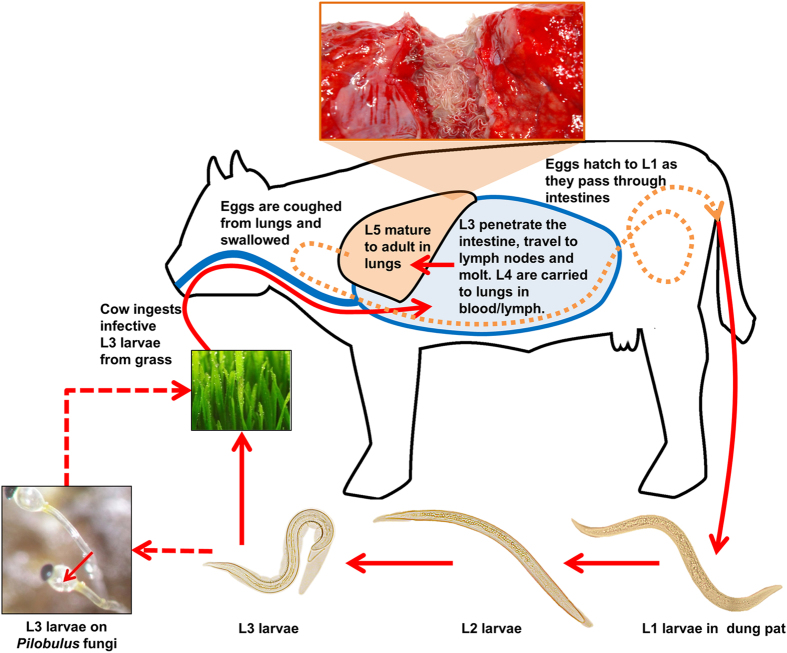
The life cycle of *Dictyocaulus viviparus.* Adult male and female worms reside in the lung. Eggs are coughed up and swallowed and hatch as they pass through the intestinal tract. Larval worms on the pasture undergo two molts to reach the infectious L3 stage, which retains its L1 and L2 cuticle as a protective sheath. L3 are ingested by grazing cattle, and parasites molt twice before reaching the lung. Further growth and development are required to become mature, reproductive adults. Photos are courtesy of Christina Strube, and the cow schematic was drawn by Bruce Rosa.

**Figure 2:**
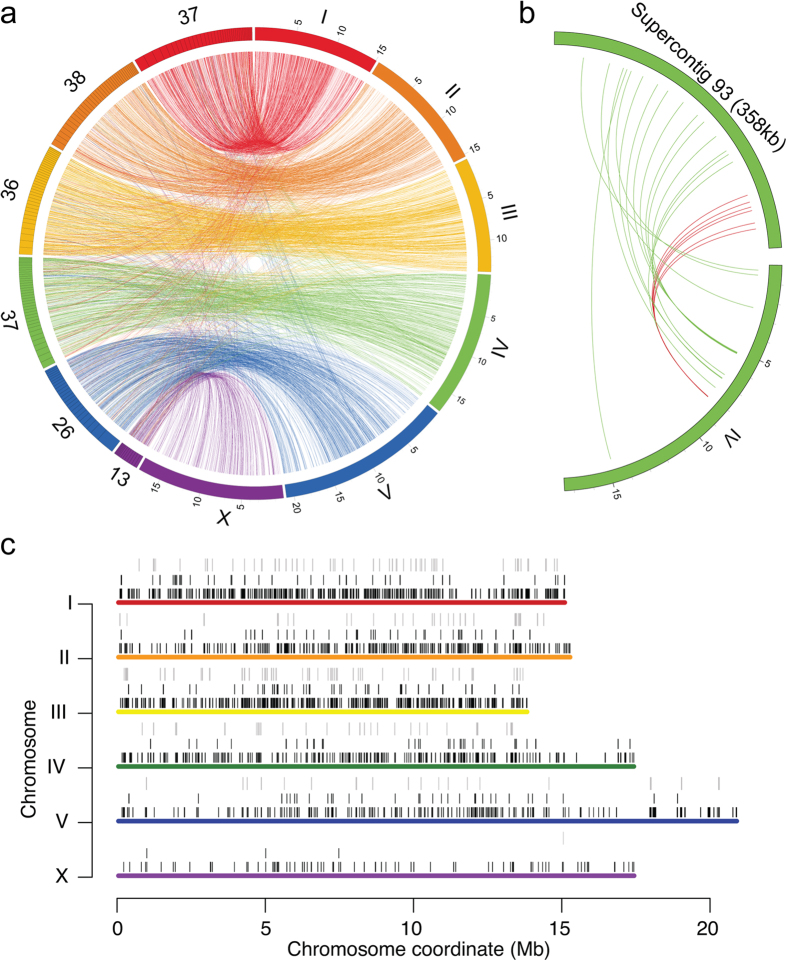
Organization of the *D. viviparus* genome compared with *C. elegans.* (**a**) A total of 187 *D. viviparus* supercontigs encode ten or more genes with orthologs in *C. elegans*. Supercontigs are colored according to their correspondence to a given *C. elegans* chromosome and ordered according to length. Lines connect *D. viviparus* genes with their orthologs in *C. elegans*. (**b**) Lines connect orthologs on *D. viviparus* supercontig-93 and *C. elegans* chromosome IV. Red lines indicate gene order conservation between the two species while green lines connect orthologs whose order is not preserved. (**c**) The relative positions of *C. elegans* operon genes (first track, black ticks), *D. viviparus* conserved (second track, black ticks) and partially conserved operon genes (third track, grey ticks) are shown on *C. elegans* chromosomes.

**Figure 3:**
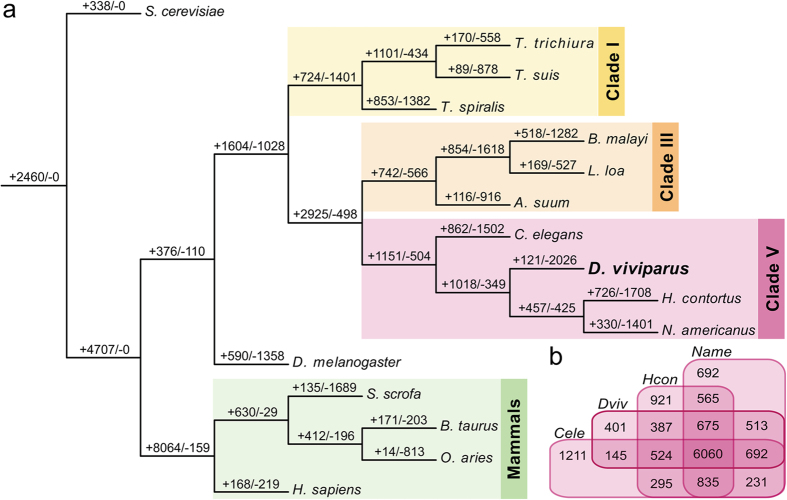
Birth and death of orthologous protein families among nematodes, hosts and outgroups. (**a**) The number of orthologous protein families gained and lost along the phylogenetic lineage of 16 species is displayed over each branch (‘+ ’ indicates gain and ‘−’ indicates loss). Clade I, III, and IV nematodes as well as their hosts and outgroups were included for comparison. (**b**) Euler diagram indicates the distribution of OPFs among Clade V nematodes, including *D. viviparus*.

Embryonated eggs are released from adult females; they are coughed up and swallowed, and hatch to L1 as they pass through the gastrointestinal tract (Fig. 1). Genes overexpressed in eggs compared with L1 were more likely to be conserved across the nematode species studied here (*P* = 5 × 10^−8^, according to OrthoMCL analysis) and were enriched for GO terms related to cell adhesion, DNA mismatch repair and signal transduction (Fig. 5b, Supplementary Table S7). GO terms related to nucleosome assembly, neuropeptide signaling and protein translation were overrepresented for genes that were overexpressed in L1 (Fig. 5b, Supplementary Table S7). Interestingly, genes involved in redox regulation appeared to be overexpressed in L1 as compared with eggs, which is opposite of the pattern reported for *H. contortus*^20^. This finding might reflect a biological difference between the two species, as *D. viviparus* eggs hatch in the microaerobic environment of the host intestine, whereas *H. contortus* eggs hatch after they have been expelled from the host into an aerobic environment.

Our results indicate that more than half of all *D. viviparus* genes were differentially transcribed between free-living and parasitic stages. GO terms related to the regulation of transcription, GPCR and steroid hormone-mediated signaling, and the transport of ions and oxygen are overrepresented among genes overexpressed in free-living larvae relative to other stages (Fig. 5b, Supplementary Table S7). These functions are similar to those enriched in *N. americanus* L3s compared with the adult stage^22^. Homeobox InterPro domains (Supplementary Table S6), typically associated with proteins involved in morphogenesis, and genes with *Wolbachia-*like sequences (*P* = 0.03) were also overrepresented in this subset of genes. Upon infection, *D. viviparus* larvae are exposed to the host immune system and begin to feed for the first time within the host. Thus, GO terms related to protein translation, redox homeostasis and energy metabolism (specifically the TCA cycle and ATP synthesis) were overrepresented among genes overexpressed in parasitic larvae (Fig. 5b, Supplementary Table S7).

We assessed sexual differentiation between immature and mature adults. Overall, clustering indicated that the transcription pattern in mature males was quite distinct from that of females and larvae (Fig. 5a), as is the case for *H. contortus*^20^. Despite obvious morphological differences between L5 males and females^34^, fewer genes were differentially transcribed between the sexes at this time point compared with mature adults (Fig. 5b; Supplementary Table S8). GO terms related to protein phosphorylation, neurotransmitter activity and proteolysis were enriched in genes overexpressed in male L5 as compared with female L5 (Fig. 5b, Supplementary Table S7), as is the major sperm protein InterPro domain (Supplementary Table S6), consistent with other parasitic nematodes^20^. GO terms related to regulation of transcription, sulfate transport, and chitin metabolism were enriched in genes overexpressed in female L5 as compared with male L5 (Fig. 5b, Supplementary Table S7). These genes may have an important role in oogenesis, given that chitin is a vital component of the nematode eggshell and that chitin metabolism genes are highly transcribed in the ovary of *Ascaris suum* compared with other tissues^35^. In contrast, GO terms related to transcriptional regulation, cell-cell adhesion, and signaling were enriched among genes overexpressed in mature females compared with mature males (Fig. 5b, Supplementary Table S7), so it is likely that these processes are related to reproduction and embryogenesis in mature females.

We specifically explored transcription in hypobiotic L5 (L5hyp) in an attempt to infer the molecular mechanism(s) responsible for maintenance of the parasite’s developmental arrest within the host animal, a process critical to the year-to-year survival of *D. viviparus* in temperate regions. Although L5hyp have undergone the final molt, they are smaller than active L5 and have an underdeveloped genital primordium^16^. Interestingly, the transcriptome profile of L5hyp was more similar to L4 than active L5 (Fig. 5a), and comparisons of L5hyp with active L5 were very similar to comparisons between L4 and active L5. A total of 217 genes were significantly overexpressed in L5hyp compared with L4 (Supplementary Table S8), and these were enriched for GO terms associated with proteolysis, response to heat, and lipid transport (Supplementary Table S7). A previous study suggested that an upregulation of transcription factors might aid in the initiation and maintenance of the hypobiotic state^16^. We identified 11 TFs among the 417 genes, which are differentially transcribed between L4 and L5hyp, although they are not significantly over-represented.

### Nematode-host interactions

Secreted and excreted proteins play a vital role in host-parasite interaction and disease. A total of 1,171 *D. viviparus* proteins contained predicted secretion signals and no transmembrane domains (Supplementary Table S3), suggesting that they may be released from the cell. This subset of proteins was significantly enriched for protease and CAP domains (Supplementary Table S6). Interestingly, the same molecular groups were shown to be overrepresented among genes with the highest non-symonymous SNP rates in our variome analysis. Increased levels of sequence variation in proteases and CAP domain proteins may help facilitate rapid adaptation to a changing host environment.

In total, 478 proteases, representing five key subclasses (175 metallo, 169 serine, 101 cysteine, 20 threonine and 13 aspartic peptidases), were found in the *D. viviparus* genome (Supplementary Table S3); 18.8% of these were predicted to be secreted. The chymotrypsin-like InterPro domain was the most significantly enriched among the putative secreted proteins (IPR001314; *P* = 4 × 10^−11^, Supplementary Table S6). Some 19% of all predicted serine proteases were considered *D. viviparus*-specific with respect to the 16 species considered in our OPF analysis, and a further 35 predicted serine proteases belonged to a chymotrypsin OPF that was significantly expanded in *D. viviparus. T. muris* serine proteases have been implicated in the degradation of intestinal mucus, but they are incapable of degrading Muc5ac, the predominant mucin of the lung^28^. Nematodes are known to induce Muc5ac expression in the host intestine, which confers a degree of protection due to structural differences in the mucus layer and direct effects on parasite viability^36^. It would be interesting to test the activity of *D. viviparus* serine proteases in a similar manner, as an ability to degrade Muc5ac may contribute to the survival of *D. viviparus* in the lung environment.

SCP/TAPS proteins (containing CAP domains) were significantly enriched among the proteins classified as *D. viviparus* specific in our OPF analysis. Some 65 *D. viviparus* proteins were predicted to contain one or more CAP domains, and 17 of these contained predicted secretion signals. SCP/TAPS proteins were abundantly represented in previous studies of *D. viviparus* and other strongylids^17^,^21^,^22^. These proteins often have extracellular endocrine or paracrine functions^37^. The crystal structure of *Na-*ASP-2, a CAP-domain protein from *N. americanus*, showed some similarity to mammalian chemokines, leading to speculation that it might serve as an agonist or ligand for mammalian chemokine receptors^38^.

### Putative drug targets

An attempt to identify putative drug targets considered Food and Drug Administration (FDA)-approved drugs with known modes of action. Most current drugs target a small number of molecular groups, including rhodopsin-like G-protein coupled receptors (GPCRs), ligand-gated ion channels (LGICs), and voltage-gated ion channels (VGICs)^30^. Rhodopsin-like GPCRs are involved in transducing extracellular signals (e.g., light, hormones and neurotransmitters) via interaction with guanine nucleotide binding proteins (G proteins). Some 147 *D. viviparus* genes were annotated as rhodopsin-like GPCRs based on Interpro domain annotations (IPR000276 and IPR017452, Supplementary Table S3). Previous studies have identified 102 LGIC and 177 VGIC genes in the *C. elegans* genome^39^. A total of 54 and 75 *D. viviparus* genes were in OPFs with *C. elegans* LGICs and VGIC’s, respectively (Supplementary Table S3). *C. elegans* encodes six glutamate gated chloride channel subunits (*glc1–4*, and *avr14–15*), some of which serve as targets for the anthelmintic ivermectin^40^. Ivermectin-sensitive *D. viviparus* is missing a *C. elegans*-specific *glc1* gene (the canonical ivermectin target) homolog, but putative orthologs were identified for *avr14–15* and *glc2–4* (Supplementary Table S9).

Protein kinases represent a major component of eukaryotic genomes and are involved in regulating a wide array of cellular activities. Kinase inhibitors have been used to treat human diseases^41^, and studies have suggested that it may be possible to re-purpose some of these drugs as anthelmintics^42^. A total of 299 kinases were identified in the *D. viviparus* genome (Supplementary Table S3), including at least one representative from each of the major kinase classes used in the search. CK1 kinases were the only class that was significantly more likely than other genes to be nematode-specific (*P* = 3 × 10^−4^).

Metabolic potential is a critical determinant governing development, growth and pathogenicity, such that metabolic enzymes might be attractive drug targets (e.g.^43^). To that end, the metabolic potential of *D. viviparus* was determined and compared with *N. americanus, C. elegans*, and the host species of the parasitic worms (*B. taurus* and *H. sapiens*). KEGG module and pathway analyses indicated that 32 modules representing 22 pathways were complete in *D. viviparus* (i.e. given the module substrates, the organism has the requisite enzymes to obtain the final products). Comparison of the modules present in the three nematodes and two mammals showed that the shared metabolic processes were consistent with their taxonomic position (Fig. 6a). The same metabolic pathway modules were determined to be complete in *D. viviparus* and the cow genome; however, completion of a metabolic module in two species does not imply that they are identical. We found three instances where *D. viviparus* and *B. taurus* employed non-orthologous enzymes to carry out the same function: modules M00003, M00048 and M00120. Figure 6b shows the module M00003 (gluconeogenesis, oxaloacetate = >fructose-6 P), which was inferred to be complete in both species, but the phosphoglycerate mutase enzyme in the parasite (K15633, conserved in nematodes and other invertebrates) is not orthologous to the host enzyme (K01837/K01834, conserved in mammals). Subtle differences such as these may provide opportunities to disrupt parasite metabolism without affecting host molecules.

Furthermore, we explored metabolic chokepoint reactions, defined as reactions that produce a unique product or consume a unique substrate. The enzymes catalyzing these reactions cannot be by-passed, such that their inhibition would halt the wider metabolic network in which they participate. Chokepoint analyses have been used to predict drug targets in several pathogenic organisms, including nematodes^22^,^43^. In total, 751 *D. viviparus* enzymes were classified as chokepoints; 308 of them had significant matches in the RSCB Protein DataBank and DrugBank; 146 of the 308 (47%) were highly transcribed in parasitic relative to free-living stages (enrichment *P* < 1 × 10^−10^), and two of them were conserved and specific for nematodes (Supplementary Table S3). One of these was DICVIV_13071, which encodes a protein with a carboxylesterase domain (type B; IPR002018). Human carboxyesterases have been implicated in xenobiotic degradation^44^, but nematode carboxyesterases have not been thoroughly characterized. The other druggable chokepoint, DICVIV_01858, encodes a homolog of *C. elegans* ACE*-3* and has a signal petide. Nematode acetylcholinesterases (including the ACE-1 homolog of *D. viviparus*^45^) may be important for parasitism due to their modulatory role on the proliferation of host epithelial cells^46^. DICVIV_01858 was only associated with 1 non-synonymous SNP in our variome analysis, reflecting a level of conservation that may indicate biological importance.

### Potential for functional genomic studies

RNAi mediated gene knockdown is an important tool for assessing gene function in some nematodes. Various RNAi protocols have been implemented in *C. elegans*, but the application of similar protocols has been met with mixed success in parasitic worms^47^. Inconsistent RNAi knockdown results may be due to differences in the RNAi effector complement present in a given species. Therefore, we matched *C. elegans* RNAi effector proteins to their orthologs in *D. viviparus* (Supplementary Table S3). Nearly half of the RNAi effector proteins appeared to be absent from *D. viviparus*; however, presence and absence is not evenly distributed over the effector classes we investigated. For instance, we identified putative orthologs of three RISC proteins and a complement of argonautes and nuclear effectors, but short interfering RNA (siRNA) amplification and spreading proteins were poorly represented. This might indicate that gene silencing is possible in *D. viviparus* if siRNAs are effectively delivered to the appropriate target tissues, but that the siRNA signals are unlikely to spread systemically as they do in *C. elegans*. Furthermore, all of the identified RNAi effector orthologs are transcribed in the stages most relevant to *in vitro* experimentation: exsheathed L3s, adult males, and adult females. Future studies will be required to assess the feasibility of RNAi in *D. viviparus*; however, the knowledge obtained here will be useful for guiding functional genomic studies.

## Conclusions

The draft genome, variome, and developmental transcriptome of *D. viviparus* provide important insights into the biology of an economically important lungworm of cattle. With its smaller genome size and gene count, the bovine lungworm clearly differs from related parasites of the gastrointestinal tract. This is the first report on genome-wide variation for any parasitic nematode, marking the onset of genetic variation mapping for *D. viviparus*. This study builds on existing knowledge of genome-wide variation in free-living nematodes such as *C. elegans*^48^ and limited, marker-based surveys of polymorphism in *D. viviparus*^31^,^32^,^33^ and other parasites, and will serve as a basis for assessing variation levels in future inter-population comparisons. In this study, we have also outlined the transcriptional changes that take place throughout the life cycle of the lungworm, with emphasis on major transitional points: hatching, host infection, sexual differentiation, and hypobiosis. We highlighted proteins that are likely to play important roles in host-parasite interactions, molecules that may be investigated as drug targets, and putative RNAi effector proteins. This work provides a solid foundation for evolutionary studies with related nematode parasites and, importantly, translational research focused on improved methods for the diagnosis, prevention, treatment, and control of dictycaulosis in cattle.

## Materials and Methods

### Ethics statement

Animal experiment protocols were approved by the ethics commission of the Lower Saxony State Office for Consumer Protection and Food Safety under reference numbers AZ 33.9–42502–06/1160 and AZ 33.9–42502–04–09/1790. All experiments were performed in accordance with relevant guidelines and regulations.

### Parasite material and nucleic acid isolation

*D. viviparus* were collected from parasite-naïve, experimentally infected Holstein-Friesian calves (*Bos taurus*) as previously described^16^. L1 were isolated from fresh, rectal faeces using the Baermann method. Isolated L1 were incubated in tap water for one and six days to develop into L2 and L3, respectively. L3 were chilled to induce hypobiosis and introduced to calves via oral infection; calves were sacrificed and mixed-sex hypobiotic L5 (<5 mm in length) were collected from the lungs 35 days post-infection^16^. Calves infected with un-chilled L3 were sacrificed at seven, 15, and 28 days post-infection for collection of L4, L5, and adults via lung perfusion. L5 and mature males and females were separated based on morphological characteristics^34^. Embryonated eggs were extracted from patent females. Precautions were taken to prevent exposure of experimental hosts to other parasite species and all specimens were morphologically to the species level. DNA and RNA isolations are described in Supplementary Methods.

### RNA-seq

Titanium fragment libraries representing *D. viviparus* eggs, mixed L1/L2, L3, mixed-sex L5, adult males, and adult females were generated and sequenced on a Genome Sequencer Titanium FLX (Roche Diagnostics, Indianapolis, IN, USA) as previously described^15^. Additionally, duplicate paired-end cDNA libraries representing *D. viviparus* eggs, L1, L2, L3, L4, mixed-sex L5, male L5, female L5, hypobiotic L5, adult males, and adult females were generated using the Illumina platform as previously described^35^. Reads were deposited in the GenBank Sequence Read Archive (SRA) under BioProject ID PRJNA72587 and in the Gene Expression Omnibus (GEO^49^,^50^) under GEO Series accession number GSE73863 (Supplementary Table S10). RNA-seq read cleaning, mapping, and transcript assembly are described in Supplementary Methods.

### Genome sequencing, assembly, and annotation

Whole genome shotgun fragment and paired-end libraries (3 kb and 8 kb insert) were constructed according to standard methods and sequenced on the Roche/454 platform^51^. While paired-end libraries were constructed from pooled males due to the amount of DNA required, the fragment library was constructed from a single adult male in order to limit genetic diversity in the contig assembly. Relevant linker and adapter sequences were trimmed, and cleaned reads were assembled with Newbler^51^. An in-house assembly improvement tool, Pygap, was used to join and extent contigs. The quality of the assembly was assessed using the CEGMA method^52^; 245 of 248 low copy CEGs were identified, and only 13% of these were present in more than one copy (as compared to 11% in the *C. elegans* genome), an indication that our assembly is (1) nearly complete and (2) that it does not contain an abundance of un-collapsed alleles. The whole-genome sequence of *D. viviparus* has been deposited in DDBJ/EMBL/GenBank under the project accession AZAF00000000 (http://www.ncbi.nlm.nih.gov/nuccore/744889955).

Repeat library generation, repeat characterization, repeat masking, and prediction of rRNAs, tRNAs, and non-coding RNAs are described in detail in the Supplementary Methods. Protein coding genes were predicted using a combination of various *ab initio* programs and the MAKER annotation pipeline^53^ which employs assembled mRNAs (i.e, Illumina cDNA assembled with Cufflinks and Roche/454 cDNA assembled with Newbler), EST (GenBank EST database), and protein evidence from the same and related species to aid in gene structure determination. A consensus gene set based on these predictions was generated using a hierarchical approach developed at The McDonnell Genome Institute^22^, and gene product naming was determined by BER (http://ber.sourceforge.net). The functional annotation (and enrichment) of protein coding genes, including comparisons to the GenBank non-redundant protein database (downloaded April 15, 2014), predicted secretion and transmembrane domains, putative proteases and inhibitors, KEGG orthologs^54^, InterPro domains and GO terms^37^, RNAi effectors^47^, kinases, drug targets, and chokepoints. Gene prediction and annotation are described in greater detail in Supplementary Methods.

### Orthology and Orthologous Protein Family Predictions

InParanoid (version 4.1^55^) was used to perform a direct comparison between *D. viviparus* and *C. elegans* proteins using the longest isoform of each *C. elegans* gene from WormBase build WS230. OrthoMCL^56^ was used to perform a broader comparison with 16 species including *D. viviparus* and the following: *Saccharomyces cerevisciae, Drosophila melanogaster, Homo sapiens, Bos taurus, Ovis aries, Sus scrofa*; *Caenorhabditis elegans, Brugia malayi, Trichonella spiralis, Ascaris suum, Haemonchus contortus, Necator americanus, Loa loa, Trichuris trichiura,* and *Trichuris suis* (see Supplementary Methods for versions). The birth and death of orthologous protein families (OPFs) among these species was predicted using Dollop (http://evolution.genetics.washington.edu/phylip/doc/dollop.html) as previously described^26^. Further analyses, including statistical enrichment, are described in Supplementary Methods.

### Identification of Operons

The known spliced leader sequences from clade V nematodes (3 SL1 and 36 SL2 sequences^57^) were used to find related trans-spliced genes in *D. viviparus* as previously described^22^ (see Supplementary Methods). Reciprocal best BLAST hits (using WU-BLAST with cutoff of 30% identity and 35 bits) between *D. viviparus* genes and 3,677 *C. elegans* operon genes (WS230^58^) were used to infer *D. viviparus* operons as previously described^22^. Operons with at least two *D. viviparus* homologs that are adjacent to each other or are separated by one neighbor were counted. For every pair of genes in every inferred operon in *D. viviparus*, Pearson’s correlation coefficient was calculated for FPKM values determined from our RNAseq data. This was compared to a “background” set of non-operon neighboring gene pairs. 5,000 pairs of genes belonging to same operon were selected at random (with replacement) and compared to 5,000 randomly selected neighboring gene pairs from the set of non-operon genes. This was also tested with 10 randomly selected instances of the background set for each operon with even more significantly different distributions.

### Genomic variation analysis

**Figure 4:**
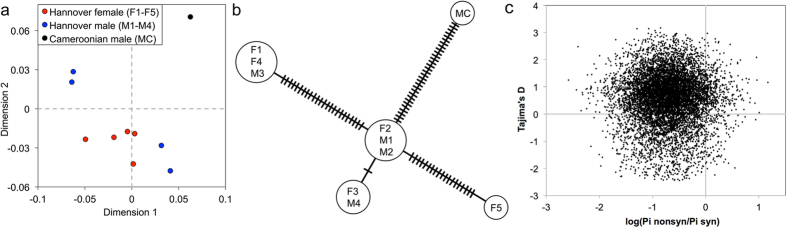
Intrapopulation genetic variability in Dictyocaulus viviparus. (**a**) Identity-by-state multidimensional scaling (IBS-MDS) plot based on autosomal SNPs displaying the genetic relationships between ten individual *Dictyocaulus viviparus* genomes. (**b**) Minimum spanning network of *Dictyocaulus viviparus* mitochondrial haplotypes based on 64 segregating SNPs. Mutational steps are represented as hatch marks. (**c**) Tajima’s D and the nonsynonymous over synonymous π ratios were calculated for each individual gene and plotted accordingly.

**Figure 5:**
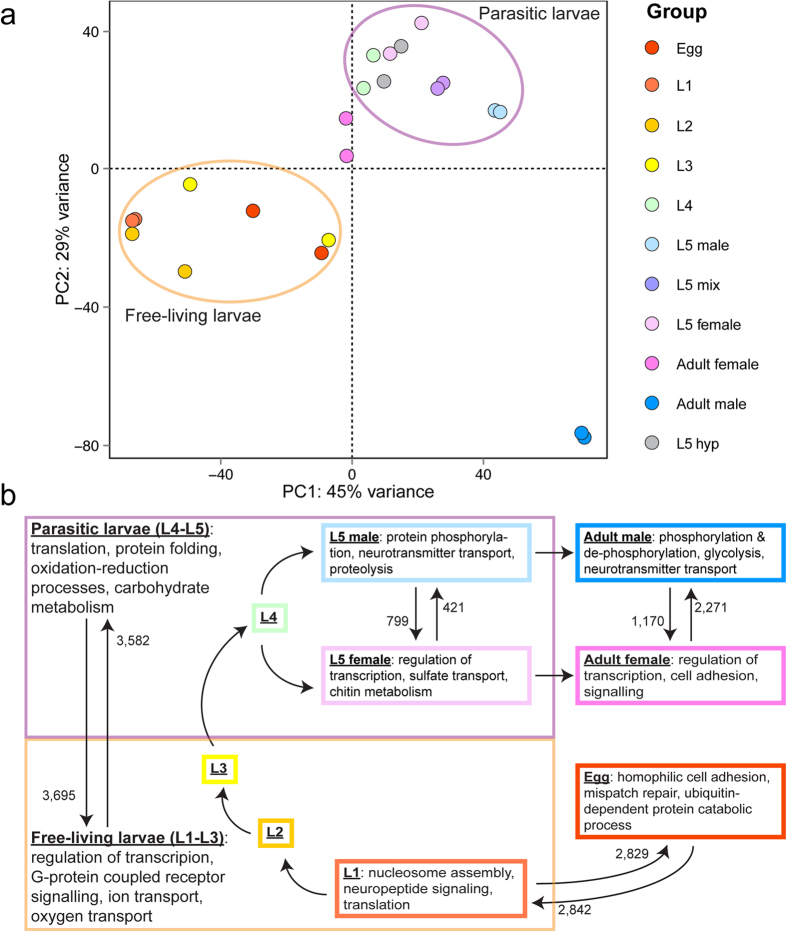
Gene transcription across the life cycle of *Dictyocaulus viviparus.* (**a**) Principal component analysis (PCA) plot indicates the clustering of RNAseq datasets (two replicates per life cycle stage) based on gene transcription. PCA indicated that 79.9% of variance was explained by the first two components (shown in the plot). (**b**) A total of 12,681 genes were differentially expressed to some degree over the course of the normal life cycle. Single arrows denote progression through the life cycle, while double arrows indicate differential gene expression comparisons. The number of genes overexpressed in a given comparison and the most enriched biological process gene ontology terms among overexpressed gene sets are indicated.

**Figure 6:**
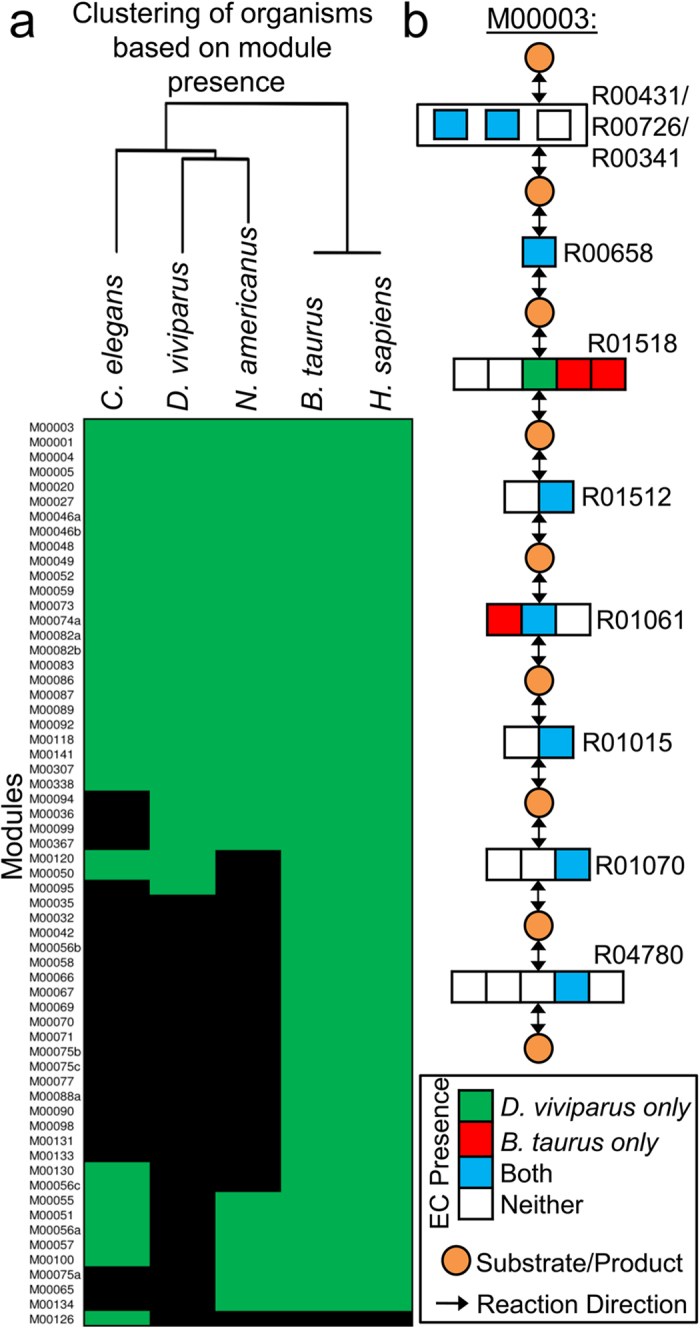
Metabolic pathway module completion in *Dictyocaulus viviparus* and other species. (**a**) Completion of KEGG metabolic pathway modules is compared between *D. viviparus*, other clade V nematodes, and relevant host species. Clustering of species based on module completeness reflects taxonomical relationships. Though there are no modules that appear to be complete in *D. viviparus* but not in its host, subtle differences do exist. (**b**) The enzymes responsible for the conversion of 2-Phospho-D-glycerate to 3-Phospho-D-glycerate (reaction R01518) in *D. viviparus* and *B. taurus* are not orthologous.

Genomic DNA was isolated from four male worms and five female worms of the DvHannover 2010 strain obtained from a single host, and paired end libraries were generated and sequenced on the Illumina HiSeq 2500 platform. Raw reads were deposited in the SRA under BioProject ID PRJNA72587 (Supplementary Table S11). Data available from the SRA representing a strain from Cameroon were also included in our analysis (SRA accession ERX364141)^24^. Reads were trimmed of relevant barcodes and adapters and aligned to the DvHannover2000 reference genome using BWA-MEM (version bwa0.7.5a, default parameters^59^) then realigned around indels using the Genome Analysis Toolkit (GATK, v.3.3.0^60^). Variants were called using GATK’s HaplotypeCaller and annotated using SnpEff (v. 3.5^61^). *F*-statistics and nucleotide diversity were computed using VCFtools (v0.1.12b)^62^. The identification of X-linked contigs (Supplementary Fig. S4), contamination filtering in HaplotypeCaller (Supplementary Fig. S5), mitochondrial SNP identification, and the production of the minimum spanning network (Fig. 4b) are all described in detail in Supplementary Methods.

To calculate nucleotide diversity separately for the nonsynonymous and synonymous sites (π_N_ and π_S_) within each gene, nonsynonymous or synonymous average pairwise differences were divided by the number of nonsynonymous or synonymous sites, respectively (see Supplementary Methods). The number of nonsynonymous or synonymous sites was determined using KaKs_Calculator 2.0^63^. Tajima’s D test^64^ was performed using VCFtools (v0.1.12b)^62^ for 5-kb sliding windows along the length of each contig. The gene-wise Tajima’s D statistic was calculated by averaging the D statistic values of all windows overlapping the gene footprint (including both exonic and intronic regions).

### Gene expression, alternative splicing and differential expression analyses

Pre-processed, paired-end, Illumina RNAseq reads were mapped onto the *D. viviparus* genome assembly with TopHat2^65^. Refcov (http://gmt.genome.wustl.edu/packages/refcov/) was used to assess the genes’ breadth of coverage based on all available RNAseq datasets; genes with ≥50% coverage by RNAseq reads were considered expressed. The number of reads associated with each feature was determined using HTSeq-Count^66^. Raw mapped read counts and fragments per kilobase per million reads mapped (FPKM) values are available through GEO^49^,^50^ (Series accession GSE73863). Differentially expressed genes were predicted using DESeq2 (version 1.4.5^67^) with an adjusted p-value cutoff of 0.1 according to established protocols^68^. Statistical enrichments are described in Supplementary Methods. RNA-Seq profiles have been deposited in Nematode.net and a browse-able genome is also available at Nematode.net and WormBase.

## Additional Information

**How to cite this article**: McNulty, S. N. *et al. Dictyocaulus viviparus* genome, variome and transcriptome elucidate lungworm biology and support future intervention. *Sci. Rep.* **6**, 20316; doi: 10.1038/srep20316 (2016).

## Supplementary Material

Supplementary Information

Supplementary Information

## Figures and Tables

**Table 1:** Features of the *Dictyocaulus viviparus* genome assembly.

Assembly length	161.0 Mb
Genome completeness	99%
Supercontigs*	7,157
Supercontig N50	225,748 bp
GC content	34.8%
Repetitive sequences	18.96%
Total protein coding genes	14,171
Average CDS length	983 bp
Average gene footprint	3,080 bp
Average exons per gene	7.1
Average exon length	119 bp
Average introns per gene	6.1
Average intron length	362 bp

*Supercontigs are defined as all scaffolds (contigs joined by inferred gaps) plus singleton contigs of >1 kb.
