# Supplementary material for: Dictyocaulus viviparus genome, variome and transcriptome elucidate lungworm biology and support future intervention
Source: Sci Rep. 2016 Feb 9;6:20316. doi: 10.1038/srep20316 (PMC4746573; doi:10.1038/srep20316)
Supplement: Supplementary Information [file srep20316-s1.pdf]

Dictyocaulus viviparus genome*,* variome and transcriptome elucidate lungworm biology and support future intervention

Samantha N. McNulty[1], Christina Strube[2], Bruce A. Rosa[1], John C. Martin[1], Rahul Tyagi[1], Young-Jun Choi[1], Qi Wang[1], Kymberlie Hallsworth-Pepin[1], Xu Zhang[1], Philip Ozersky[1], Richard K. Wilson[1], Paul W. Sternberg[3], Robin B. Gasser[4], Makedonka Mitreva[1, 5]*

[1] The McDonnell Genome Institute, Washington University in St Louis, MO 63108, USA

[2] Institute for Parasitology, University of Veterinary Medicine Hannover, Hannover 30559, Germany

[3] HHMI, Division of Biology, California Institute of Technology, Pasadena, CA 91125, USA

[4] Faculty of Veterinary and Agricultural Sciences, The University of Melbourne, Victoria 3010, Australia

[5] Division of Infectious Diseases, Department of Medicine, Washington University School of Medicine, St. Louis, MO 63110, USA

*Corresponding author: Makedonka Mitreva, The McDonnell Genome Institute, Washington University in St Louis, 4444 Forest Park Avenue, St. Louis, MO 63108, USA. Email: (mmitreva@genome.wustl.edu)

Supplementary Figures

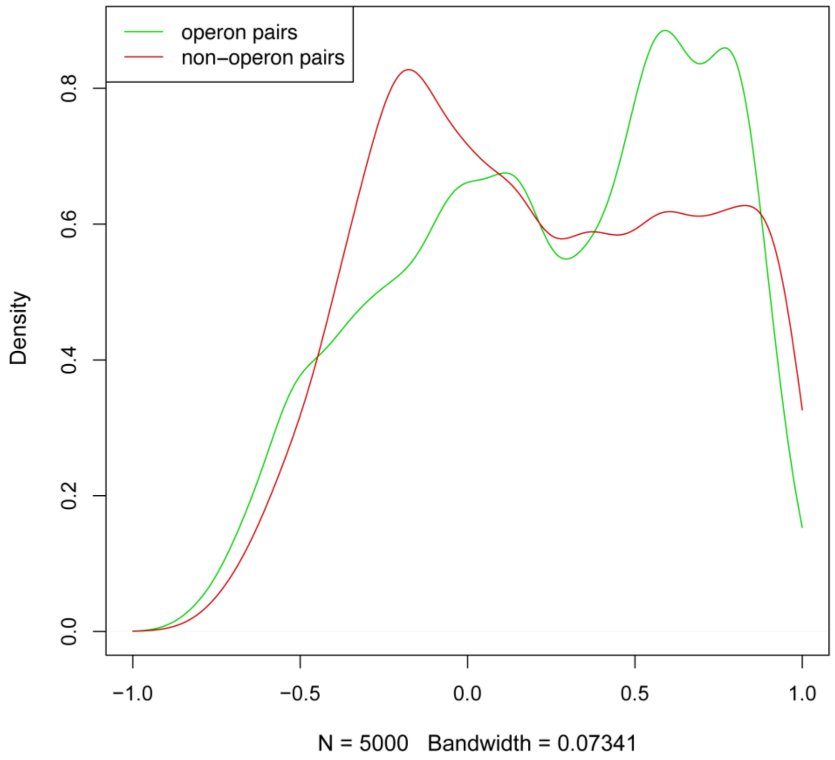

Supplementary Figure S1. Co-transcription of predicted operon and non-operon genes
The mean Pearson's correlation coefficient for every pair of genes in an operon in *D. viviparus* are plotted (green) against that of a pseudo-operon of the same size, consisting of randomly selected genes (red). The correlation coefficients for genes in predicted operons are significantly higher than for background.

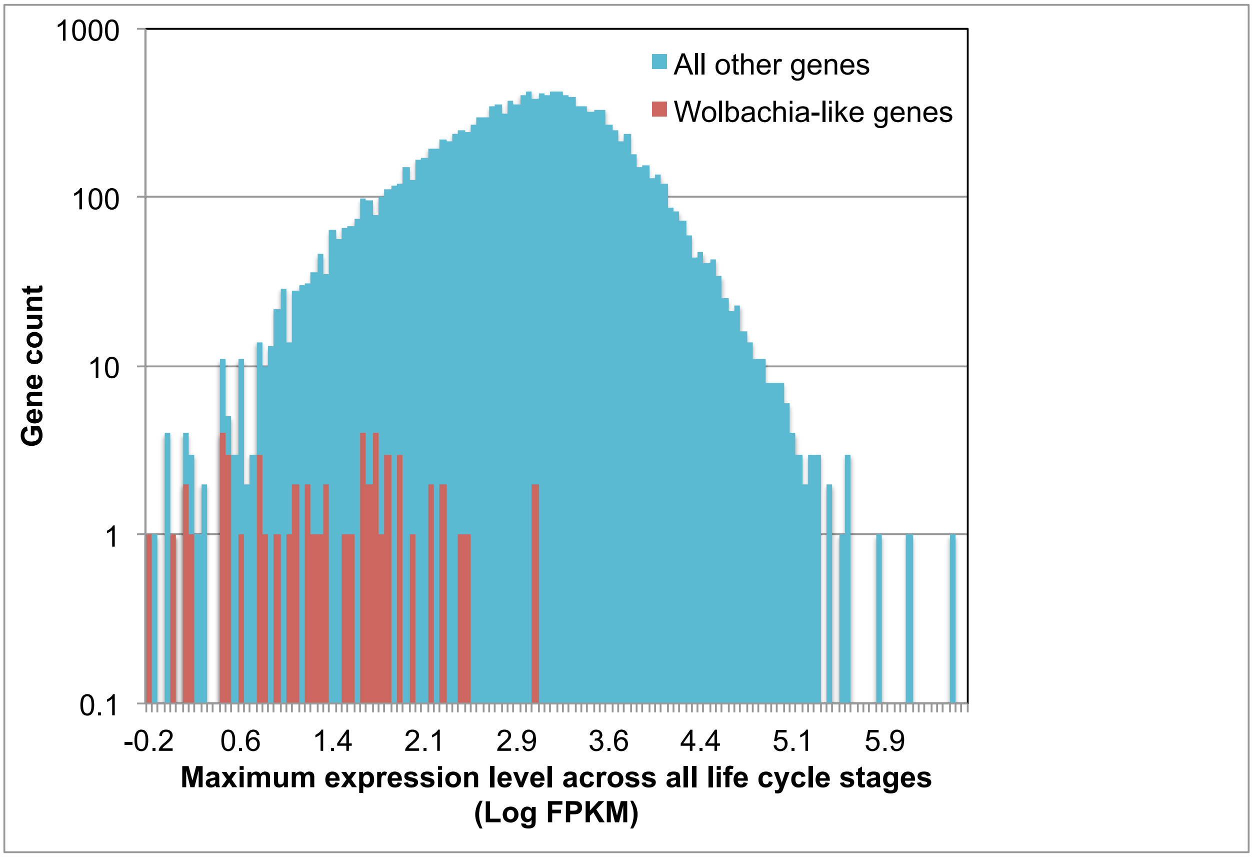

Supplementary Figure S2. Maximum expression (FPKM) of *Wolbachia*-like and other genes throughout all life cycle stages of *D. viviparus*

The maximum expression level (given in fragments per kilobase per million reads mapped, FPKM) for each gene was determined and potted to compare the expression of *Wolbachia*-like and non-*Wolbachia*-like genes. *Wolbachia*-like genes were transcribed at low levels compared to other genes (average peak expression of 77.7 FPKM compared to 3,356.6 FPKM for other genes; $P < 10^{-10}$, T-test using log-scale FPKM values).

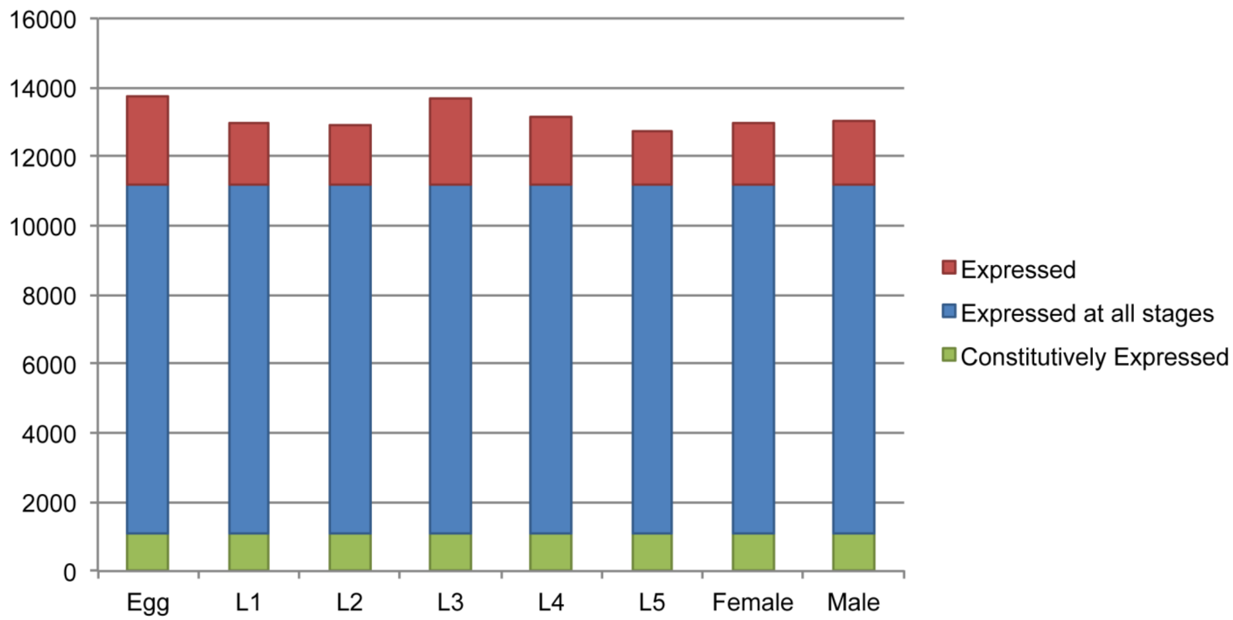

Supplementary Figure S3. Gene expression throughout the life cycle of *Dictyocaulus viviparus*

The number of genes with detectable expression (≥50% breadth of coverage with RNAseq reads) at each life cycle stage is indicated on the graph. 11,179 of the 14,171 inferred genes (79% of all genes) were transcribed, to some degree, in every life cycle stage. Of these, 1,102 showed no statistically significant variation in expression level over the course of the life cycle and were considered to be constitutively expressed.

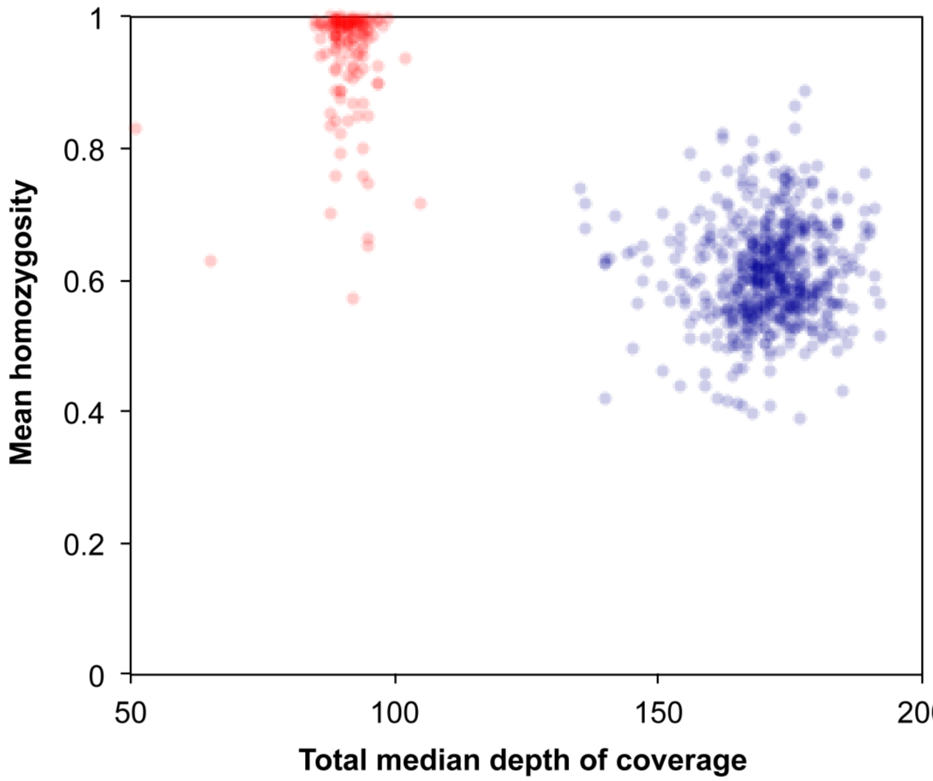

Supplementary Figure S4. Identification of X-linked and autosomal chromosome contigs

Autosomal (blue; n=563; combined length 119,815,143 bp) and X-linked (red; n=130; combined length 14,366,088 bp) contigs (> 50kb) were identified based on the contig-wise mean homozygosity and total median depth of coverage in male samples.

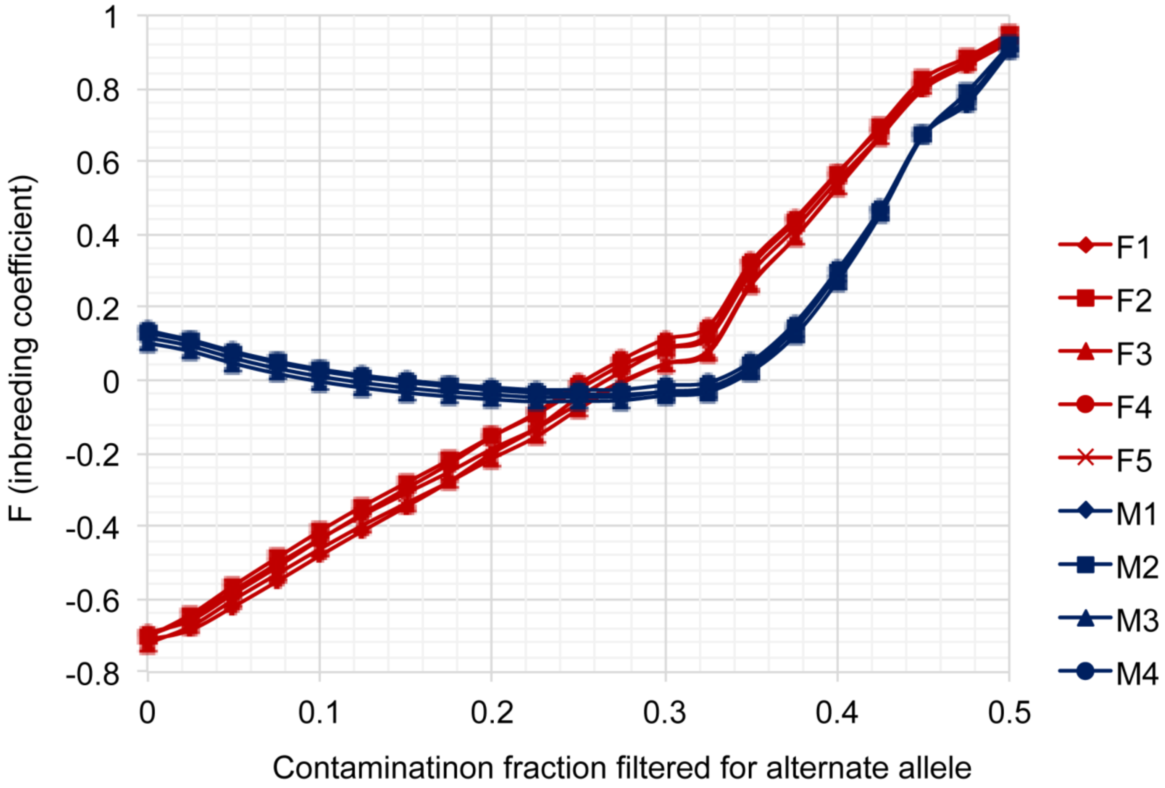

Supplementary Figure S5. Impact of contamination filtering in HaplotypeCaller on heterozygosity

Fractions of reads for each alternate allele were removed through biased down-sampling, and the observed level of heterozygosity in the resultant genotype call set was assessed relative to the expected value under Hardy-Weinberg equilibrium.

Supplementary Methods

Parasite material and nucleic acid isolation

D. viviparus samples from various life cycle stages were collected from parasite naïve, experimentally infected Holstein-Friesian calves (*Bos taurus*) as previously described[1,2]. First stage larvae (L1) were isolated from cow feces collected directly from the cow rectum using the Baermann method. For development into second and third stage larvae (L2, L3), L1 were incubated in tap water for one and six days, respectively. Isolated L3 were chilled to induce hypobiosis and introduced to calves via oral infection; calves were sacrificed and mixed-sex hypobiotic L5 (<5mm in length) were collected from the lungs 35 days post-infection[2]. Calves infected with un-chilled L3 were sacrificed at seven, 15 and 28 days post infection for collection of L4, L5 and adults via lung perfusion. L5 and adult males and females were separated based on morphological characteristics[3]. Embryonated eggs were extracted from patent females. Precautions were taken to prevent exposure of experimental hosts to other parasite species and This included the use of young calves that had never been subjected to pasture, screening cows for parasitic infection prior to use, housing cows in stables with paved outdoor access (rather than pasture), feeding on hay and concentrate rather than grass, etc. *D. viviparus* adults and larvae are readily distinguishable from other nematodes basted on morphology and movement patterns, and all *D. viviparus* specimens were subjected to morphological examination prior to use in experiments.

Genomic DNA for the reference genome was isolated from adult male worms (DvHannover2000 strain) using a NucleoSpin Tissue DNA isolation kit according to the manufacturer's recommendations (Machery-Nagel, Düren, Germany). For Roche/454 cDNA libraries, individual lungworm stages were homogenized in 5.5M GIT buffer (5.5M guanidinium thiocyanate, 1 M sodium citrate, pH 7.0) using the Precellys® 24 tissue homogenizer (Peqlab Biotechnologie, Erlangen, Germany), and mRNA was isolated from the homogenate using the Illustra QuickPrep *Micro* mRNA Purification Kit (GE Healthcare, Buckinghamshire, UK)[1]. For Illumina cDNA libraries, individual lungworm stages were homogenized in 600μL RLT-buffer (RNeasy® Mini Kit, Qiagen, Hilden, Germany) and 6μL 2-mercaptoethanol (Roth, Karlsruhe, Germany) using the Precellys® 24 tissue homogenizer (Peqlab Biotechnologie), and total RNA was isolated from the homogenate using RNeasy® Mini Kit (Qiagen) according the manufacturer's

suggested protocol, including the optional on-column DNase digest. The integrity and quantity of nucleic acid samples were assessed using the Bioanalyzer 2100 (Agilent Technologies, Cedar Creek, Texas).

cDNA sequencing on the Roche/454 platform

Titanium fragment libraries representing *D. viviparus* eggs, mixed L1/L2, L3, mixed-sex L5, adult males, and adult females were generated and sequenced on a Genome Sequencer Titanium FLX (Roche Diagnostics, Indianapolis, IN, USA) as previously described[4,5]. Raw reads were deposited in the GenBank sequence read archive (SRA) under BioProject PRJNA72587 (Supplementary Table S10). Following analytical processing[6], reads were assembled using Newbler, invoking the cDNA-specific assembly option as previously described[7].

cDNA sequencing on the Illumina platform

Duplicate paired-end cDNA libraries representing *D. viviparus* eggs, L1, L2, L3, L4, mixed-sex L5, male L5, female L5, hypobiotic L5, adult males, and adult females were generated as previously described using standard protocols[8]. Raw reads were deposited in the GenBank Sequence Read Archive under BioProject ID PRJNA72587 and in Gene Expression Omnibus (GEO) under Series accession number GSE73863 (Supplementary Table S10). Adapter trimming, sequence quality trimming, length filtering, complexity filtering, and contaminant filtering were performed as previously described[9]. The remaining high-quality RNAseq reads were aligned to the genome assembly using Tophat2[10] (version 2.0.8, default parameters) and assembled into full-length transcripts with Cufflinks (version 2.1.1[11]) using an established protocol[12].

Genome sequencing, assembly, and annotation

Whole genome shotgun fragment and paired-end libraries (3kb and 8kb insert) were constructed according to standard methods and sequenced on the Roche/454 platform[13]. Sequencing coverage was estimated at 12.3x, 21.7x and 4.4x for the fragment, 3kb, and 8kb insert libraries, respectively. Relevant linker and adapter sequences were trimmed, and cleaned reads were assembled with Newbler[13]. An in-house assembly improvement tool, Pygap, was used to join and extent contigs using unassembled

Illumina reads when possible. A repeat library was generated using Repeatmodler (http://repeatmasker.org), and repeats were characterized by screening against Repbase (April 2014) with CENSOR (version 4.2.29)[14,15]. Ribosomal RNA genes were identified using RNAmmer[16], and transfer RNA genes were identified using tRNAscan-SE[17]. Other non-coding RNAs (e.g., microRNAs) were identified by sequence homology searches against the Rfam database[18]. Repeats and predicted non-protein-coding RNAs were masked with RepeatMasker (http://repeatmasker.org).

Protein coding genes were predicted using a combination of the *ab initio* programs Snap[19], Fgenesh[20], and Augustus[21] and the MAKER annotation pipeline[22] which employs assembled mRNAs (i.e, Illumina cDNA assembled with Cufflinks and Roche/454 cDNA assembled with Newbler), EST (GenBank EST database), and protein evidence from the same and related species to aid in gene structure determination and modification. A consensus gene set based on these predictions was generated using a hierarchical approach developed at The Genome Institute[23], and gene product naming was determined by BER (http://ber.sourceforge.net). An extended annotation of these predicted genes is available at nematode.net/Dviviparus_genome.html and will be referred to in this manuscript as NN table.

Building orthologous protein family groups with related species

InParanoid (version 4.1)[24,25] was used to perform a direct comparison between *D. viviparus* and *C. elegans* proteins using the longest isoform of each *C. elegans* gene from WormBase build WS230. OrthoMCL[26] was used to perform a broader comparison with 16 species, including *D. viviparus* and the following: *Saccharomyces cerevisciae*, *Drosophila melanogaster*, and *Homo sapiens* from Ensembl release 67[27]; *Bos taurus* (release 100)*, Ovis aries* (release 100), and *Sus scrofa* (release 103) from Genbank[28]; *Caenorhabditis elegans* and *Brugia malayi* WormBase release 230[29]; *Trichonella spiralis*[30]; *Ascaris suum*[31]; *Haemonchus contortus*[32]; *Necator americanus*[23]; *Loa loa*[33]; *Trichuris trichiura* version 2.0 from the Helminth Genomes Initiative[34]; *Trichuris suis* from an in-house genome sequencing project. In applicable cases, the longest protein isoform was used as a representative for alternatively spliced genes in the OrthoMCL analysis. The birth and death of orthologous protein families (OPFs) among these species was predicted using Dollop (http://evolution.genetics.washington.edu/phylip/doc/dollop.html) as previously described[35]. Genes were considered *D. viviparus*-specific if they were not orthologous to any

genes in the 15 other species used in this analysis. OPFs containing orthologs from *D. viviparus* and at least one other nematode were subjected to a binomial distribution test to determine enrichment of *D. viviparus* orthologs with the expected (background) percentage being the proportion of genes from the genome belonging to the OPF (averaged for each other nematode in the OPF), the number of "successes" being the number of *D. viviparus* genes in the OPF, and the number of "trials" being the total number of *D. viviparus* genes (14,171). This statistic is non-parametric, and the P values were corrected using FDR population correction[36].

Prediction of *D. viviparus* operons

The known spliced leader sequences from clade V nematodes (3 SL1 and 36 SL2 sequences[37]) were used to find related trans-spliced genes in *D. viviparus* as previously described[23]. The RNAseq reads that satisfied the following criteria for similarity to known SL1 and SL2 sequences were considered to be sourced from a gene trans-spliced with a *D. viviparus* spliced leader sequence:

1. A hit was reported by blat, using the options '-tileSize=6 -oneOff=1 -minScore=12'. Matches on either strand were considered hits.

2. The match on the target sequence (RNA-Seq read) started, at most, 0 (for SL1) or 8 (for SL2) bases from either end.

3. The match on the query (the known SL sequence) started at most 2 (for SL1) or 8 (for SL2) bases from the 5' end.

4. At most 1 (for SL1) or 2 (for SL2) mismatches were allowed.

5a. For SL1, no gaps were allowed within the aligned part of the sequence.

5b. For SL2, the aligned part was to be at most 8 bases shorted than the SL2 sequence length (e.g. for a 22 base SL2, any read with aligned region smalled than 14 bases is rejected).

6. For SL2, at most a single gap of length 1 base was allowed on either the query or the target.

The reads thus identified were then aligned to the Cufflinks-assembled consensus transcripts and only those cases where the corresponding gene model is on the same strand as the putative SL sequence identified were considered to be potentially SL-trans-spliced genes.

Reciprocal best BLAST hits (using WU-BLAST with cutoff of 30% identity and 35 bits) between *D. viviparus* genes and 3,677 *C. elegans* operon genes (WS230)[38] were used to infer *D. viviparus* operons as previously described[23]. Operons with at least two *D. viviparus* homologs that are adjacent to each other or are separated by one neighbor were counted. For every pair of genes in every inferred operon in *D. viviparus*, Pearson's correlation coefficient was calculated for FPKM values determined from our RNAseq data. This was compared to a "background" set of non-operon neighboring gene pairs. 5000 pairs of genes belonging to same operon were selected at random (with replacement) and compared to 5000 randomly selected neighboring gene pairs from the set of non-operon genes. This was also tested with 10 randomly selected instances of the background set for each operon with even more significantly different distributions.

Functional annotation of protein coding genes

Inferred protein sequences were compared to known protein sequences by BLASTP[39] against the GenBank non-redundant protein database (NR, downloaded April 15, 2014). Transmembrane domains and classical secretion peptides were predicted using Phobius[40,41], and non-classical secretion signals were predicted using SecretomeP[42]. Putative proteases and inhibitors were identified and classified using the online MEROPS peptidase database server (release 9.11)[43]. Proteins were assigned to KEGG orthologous groups, pathways and pathway modules using KEGGscan[44] with KEGG release 68[45]. Associations with InterPro protein domains and Gene Ontology (GO) classifications were inferred using InterProScan[46-48]. InterPro domain enrichment for gene sets was determined (for each domain represented in at least 5 genes) using a non-parametric binomial distribution test with the expected (background) percentage being the proportion of genes in the genome containing the InterPro domain, the number of "successes" being the number of genes in the target set containing the InterPro domain, and the number of "trials" being the total number of genes in the target set. A 0.01 p value cutoff was used for significance (after Benjamini-Hochberg false-discovery-rate (FDR) population correction for the total number of domains)[36]. Functional enrichment of GO terms related to particular subsets of proteins was calculated using FUNC[49] with an adjusted p-value cutoff of 0.01. *C. elegans* RNAi effector proteins were reported previously[50]. The longest isoform of each *C. elegans* RNAi effector protein was taken from

WormBase release WS230 and matched to *D. viviparus* orthologs by inParanoid or by best bi-directional BLASTP match (e-value < $1e^{-05}$).

C. elegans proteins were screened against the collection of kinase domain models in Kinomer[51,52]. Custom score thresholds were applied for each kinase group and adjusted until an hmmpfam search[53] came as close as possible to identifying the known *C. elegans* kinases. The same procedure and cutoffs were applied to the *D. viviparus* proteins to identify kinases as previously described[23,30].

High confidence sets of enzymes available to the 5 species were obtained by using local version of KAAS server[54], with the bit-score threshold set at 35. modDFS was then used to find which of the nematode-relevant modules were strictly complete in the species[55]. Chokepoints of KEGG metabolic pathways were defined as a reaction that either consumes a unique substrate or produces a unique product[56]. The reaction database from KEGG v65[45] was used to identify chokepoints as previously described[57]. *D. viviparus* proteins were assigned EC numbers based on the output of KEGGscan (described above). Protein Data Bank[58] and DrugBank[59] were identified by WU-BLAST[60] against database sequences using a cutoff of 30% sequence identity over 75% of the length of the query.

Genomic variation analysis

Genomic DNA was isolated from four male worms and five female worms of the DvHannover 2010 strain obtained from a single host, and paired end libraries were generated and sequenced on the Illumina HiSeq 2500 platform. Raw reads were deposited in the SRA under BioProject ID PRJNA72587 (Supplementary Table S11). Data available from the SRA representing a strain from Cameroon were also included in our analysis (SRA accession ERX364141)[61]. Relevant barcodes and adapters were trimmed, and reads of less than 60bp in length and/or with uncalled bases were discarded using Flexbar[62]. Remaining reads were aligned to the DvHannover2000 reference genome using BWA-MEM (version bwa0.7.5a, default parameters[63]). Duplicate reads were marked for removal using Picard tools (http://broadinstitute.github.io/picard/). Reads were realigned around indels using the Genome Analysis Toolkit (GATK, v.3.3.0)[64], and variants were called using GATK's HaplotypeCaller. High quality SNPs were obtained using GATK's VariantFiltration with the following set of filters: DP (maximum depth) > 949;

QD (variant confidence divided by the unfiltered depth of non-reference samples) < 2.0; FS (Phred-scaled p-value using Fisher's Exact Test to detect strand bias in the reads) > 60.0; MQ (Root Mean Square of the mapping quality of the reads across all samples) < 40.0; MQRankSum (Mann-Whitney Rank Sum Test for mapping qualities) < -12.5; ReadPosRankSum (Mann-Whitney Rank Sum Test for the distance from the end of the read for reads with the alternate allele) < -8.0. Annotated SNPs were classified according to their effect based on their genomic location context using SnpEff (v. 3.5)[65].

Contigs predicted to represent the X chromosome (i.e., hemizygous loci) were identified based on contig-wise mean homozygosity and total median depth of coverage in male samples (Supplementary Fig. S4). Using SNPs located on putatively autosomal contigs, sample relationships were examined by multidimensional scaling (MDS) analysis based on pairwise identity-by-state (IBS) distance in PLINK[66]. In order to minimize the effect of excess heterozygosity in female samples (due to embryonic DNA of non-maternal origin) on MDS clustering, variants were called using HaplotypeCaller with the "-contamination" option that invokes removal of bases supporting putative variants. The contamination fraction parameter was set at 0.25, based on the restoration to the expected heterozygosity under Hardy–Weinberg equilibrium, as judged by F_{IS} (inbreeding coefficient) (Supplementary Fig. S5). *F*-statistics and nucleotide diversity were computed using VCFtools (v0.1.12b)[67]. Mitochondrial SNPs were identified using an identical procedure to that outlined above for the nuclear genomic variants except that the reference alignment was generated using a previously published mitochondrial genome (GenBank accession NC_019810). SNPs were called using GATK's HaplotypeCaller with the -ploidy argument set to 1. A minimum spanning network[68] was constructed for the mitochondrial haplotypes in PopART (http://popart.otago.ac.nz) using segregating SNPs with no missing genotype calls.

To calculate nucleotide diversity separately for the nonsynonymous and synonymous sites (π_N and π_S) within each gene, nonsynonymous or synonymous average pairwise differences were divided by the number of nonsynonymous or synonymous sites, respectively.. The number of nonsynonymous or synonymous sites was determined using KaKs_Calculator 2.0[69]. Tajima's D test[70] was performed using VCFtools (v0.1.12b)[67] for 5-kb sliding windows along the length of each contig. The gene-wise Tajima's D statistic was calculated by averaging the D statistic values of all windows overlapping the gene footprint (including both exonic and intronic regions).

Gene expression, alternative splicing and differential expression analyses

Pre-processed, paired-end, Illumina RNAseq reads were mapped onto the *D. viviparus* genome assembly with Tophat2[10] using the gff annotation file to guide alignments. Refcov[71] was used to assess the genes' breadth of coverage based on all available RNAseq datasets, and genes showing ≥50% breadth of coverage were characterized as expressed. The number of reads associated with each feature was determined using HTSeq-Count[72]. Mapped read counts and fragments per kilobase per million reads mapped (FPKM) values are available through GEO (Series accession GSE73863). Differentially expressed genes were predicted using DESeq2 (version 1.4.5[73]) with an adjusted p-value cutoff of 0.1 according to established protocols[74]. Statistically enriched InterPro domains and GO terms were determined as previously described. Over-representation of genes from each orthologous group classification (e.g., nematode-conserved, *D. viviparus*-specific, etc.) among sets of over-expressed genes was tested using a Binomial distribution test, with the expected (background) percentage being the proportion of genes from the genome belonging to the orthologous group classification, the number of "successes" being the number of overexpressed genes of interest belonging to the orthologous group classification, and the number of "trials" being the total number of overexpressed genes of interest. This statistic is non-parametric, and the *P* values were corrected using FDR population correction[36]. This test was also used in the same way to test enrichment for *Wolbachia*-like genes and genes grouped based on Tajima's D and π_N/π_S values.

References

1 Strube, C., Buschbaum, S., Wolken, S. & Schnieder, T. Evaluation of reference genes for quantitative real-time PCR to investigate protein disulfide isomerase transcription pattern in the bovine lungworm *Dictyocaulus viviparus*. *Gene* **425**, 36-43, doi:10.1016/j.gene.2008.08.001 (2008).

2 Laabs, E. M., Schnieder, T. & Strube, C. Transcriptional differences between hypobiotic and non-hypobiotic preadult larvae of the bovine lungworm *Dictyocaulus viviparus*. *Parasitology research* **110**, 151-159, doi:10.1007/s00436-011-2464-7 (2012).

3 Laabs, E. M., Schnieder, T. & Strube, C. In vitro studies on the sexual maturation of the bovine lungworm *Dictyocaulus viviparus* during the development of preadult larvae to adult worms. *Parasitology research* **110**, 1249-1259, doi:10.1007/s00436-011-2622-y (2012).

4 Wang, Z. *et al.* Characterizing *Ancylostoma caninum* transcriptome and exploring nematode parasitic adaptation. *BMC genomics* **11**, 307, doi:10.1186/1471-2164-11-307 (2010).

5 Cantacessi, C. *et al.* Deep insights into *Dictyocaulus viviparus* transcriptomes provides unique prospects for new drug targets and disease intervention. *Biotechnology advances* **29**, 261-271, doi:10.1016/j.biotechadv.2010.11.005 (2011).

6 Mitreva, M. & Mardis, E. R. Large-scale sequencing and analytical processing of ESTs. *Methods Mol Biol* **533**, 153-187, doi:10.1007/978-1-60327-136-3_8 (2009).

7 Abubucker, S., McNulty, S. N., Rosa, B. A. & Mitreva, M. Identification and characterization of alternative splicing in parasitic nematode transcriptomes. *Parasites & vectors* **7**, 151, doi:10.1186/1756-3305-7-151 (2014).

8 Rosa, B. A., Jasmer, D. P. & Mitreva, M. Genome-Wide Tissue-Specific Gene Expression, Co-expression and Regulation of Co-expressed Genes in Adult Nematode *Ascaris suum*. *PLoS Negl Trop Dis* **8**, e2678, doi:10.1371/journal.pntd.0002678 (2014).

9 McNulty, S. N. *et al.* Systems biology studies of adult paragonimus lung flukes facilitate the identification of immunodominant parasite antigens. *PLoS neglected tropical diseases* **8**, e3242, doi:10.1371/journal.pntd.0003242 (2014).

10 Kim, D. *et al.* TopHat2: accurate alignment of transcriptomes in the presence of insertions, deletions and gene fusions. *Genome biology* **14**, R36, doi:10.1186/gb-2013-14-4-r36 (2013).

11 Trapnell, C. *et al.* Transcript assembly and quantification by RNA-Seq reveals unannotated transcripts and isoform switching during cell differentiation. *Nature biotechnology* **28**, 511-515, doi:10.1038/nbt.1621 (2010).

12 Trapnell, C. *et al.* Differential gene and transcript expression analysis of RNA-seq experiments with TopHat and Cufflinks. *Nature protocols* **7**, 562-578, doi:10.1038/nprot.2012.016 (2012).

13 Margulies, M. *et al.* Genome sequencing in microfabricated high-density picolitre reactors. *Nature* **437**, 376-380, doi:10.1038/nature03959 (2005).

14 Jurka, J. *et al.* Repbase Update, a database of eukaryotic repetitive elements. *Cytogenetic and genome research* **110**, 462-467, doi:10.1159/000084979 (2005).

15 Kohany, O., Gentles, A. J., Hankus, L. & Jurka, J. Annotation, submission and screening of repetitive elements in Repbase: RepbaseSubmitter and Censor. *BMC bioinformatics* **7**, 474, doi:10.1186/1471-2105-7-474 (2006).

16 Lagesen, K. *et al.* RNAmmer: consistent and rapid annotation of ribosomal RNA genes. *Nucleic acids research* **35**, 3100-3108, doi:10.1093/nar/gkm160 (2007).

17 Lowe, T. M. & Eddy, S. R. tRNAscan-SE: a program for improved detection of transfer RNA genes in genomic sequence. *Nucleic acids research* **25**, 955-964 (1997).

18 Griffiths-Jones, S., Bateman, A., Marshall, M., Khanna, A. & Eddy, S. R. Rfam: an RNA family database. *Nucleic acids research* **31**, 439-441 (2003).

19 Korf, I. Gene finding in novel genomes. *BMC bioinformatics* **5**, 59, doi:10.1186/1471-2105-5-59 (2004).

20 Salamov, A. A. & Solovyev, V. V. Ab initio gene finding in *Drosophila* genomic DNA. *Genome research* **10**, 516-522 (2000).

21 Stanke, M., Diekhans, M., Baertsch, R. & Haussler, D. Using native and syntenically mapped cDNA alignments to improve de novo gene finding. *Bioinformatics* **24**, 637-644, doi:10.1093/bioinformatics/btn013 (2008).

22 Cantarel, B. L. *et al.* MAKER: an easy-to-use annotation pipeline designed for emerging model organism genomes. *Genome research* **18**, 188-196, doi:10.1101/gr.6743907 (2008).

23 Tang, Y. T. *et al.* Genome of the human hookworm *Necator americanus*. *Nature genetics*, doi:10.1038/ng.2875 (2014).

24 O'Brien, K. P., Remm, M. & Sonnhammer, E. L. Inparanoid: a comprehensive database of eukaryotic orthologs. *Nucleic acids research* **33**, D476-480, doi:10.1093/nar/gki107 (2005).

25 Remm, M., Storm, C. E. & Sonnhammer, E. L. Automatic clustering of orthologs and in-paralogs from pairwise species comparisons. *Journal of molecular biology* **314**, 1041-1052, doi:10.1006/jmbi.2000.5197 (2001).

26 Li, L., Stoeckert, C. J., Jr. & Roos, D. S. OrthoMCL: identification of ortholog groups for eukaryotic genomes. *Genome research* **13**, 2178-2189, doi:10.1101/gr.1224503 (2003).

27 Kinsella, R. J. *et al.* Ensembl BioMarts: a hub for data retrieval across taxonomic space. *Database : the journal of biological databases and curation* **2011**, bar030, doi:10.1093/database/bar030 (2011).

28 Benson, D. A. *et al.* GenBank. *Nucleic acids research* **42**, D32-37, doi:10.1093/nar/gkt1030 (2014).

29 Harris, T. W. *et al.* WormBase 2014: new views of curated biology. *Nucleic acids research*, doi:10.1093/nar/gkt1063 (2013).

30 Mitreva, M. *et al.* The draft genome of the parasitic nematode *Trichinella spiralis*. *Nature genetics* **43**, 228-235, doi:10.1038/ng.769 (2011).

31 Jex, A. R. *et al.* *Ascaris suum* draft genome. *Nature* **479**, 529-533, doi:10.1038/nature10553 (2011).

32 Laing, R. *et al.* The genome and transcriptome of *Haemonchus contortus*, a key model parasite for drug and vaccine discovery. *Genome biology* **14**, R88, doi:10.1186/gb-2013-14-8-r88 (2013).

33 Desjardins, C. A. *et al.* Genomics of *Loa loa*, a *Wolbachia*-free filarial parasite of humans. *Nature genetics* **45**, 495-500, doi:10.1038/ng.2585 (2013).

34 Holroyd, N. & Sanchez-Flores, A. Producing parasitic helminth reference and draft genomes at the Wellcome Trust Sanger Institute. *Parasite Immunol.* **34**, 100-107, doi:10.1111/j.1365-3024.2011.01311.x (2012).

35 Wang, Z., Zarlenga, D., Martin, J., Abubucker, S. & Mitreva, M. Exploring metazoan evolution through dynamic and holistic changes in protein families and domains. *BMC evolutionary biology* **12**, 138, doi:10.1186/1471-2148-12-138 (2012).

36 Benjamini, Y. & Hochberg, Y. Controlling the False Discovery Rate: A Practical and Powerful Approach to Multiple Testing. *Journal of the Royal Statistical Society. Series B (Methodological)* **57**, 289-300, doi:10.2307/2346101 (1995).

37 Guiliano, D. B. & Blaxter, M. L. Operon conservation and the evolution of trans-splicing in the phylum Nematoda. *PLoS genetics* **2**, e198, doi:10.1371/journal.pgen.0020198 (2006).

38 Blumenthal, T. *et al.* A global analysis of *Caenorhabditis elegans* operons. *Nature* **417**, 851-854, doi:10.1038/nature00831 (2002).

39 Camacho, C. *et al.* BLAST+: architecture and applications. *BMC bioinformatics* **10**, 421, doi:10.1186/1471-2105-10-421 (2009).

40 Kall, L., Krogh, A. & Sonnhammer, E. L. A combined transmembrane topology and signal peptide prediction method. *Journal of molecular biology* **338**, 1027-1036, doi:10.1016/j.jmb.2004.03.016 (2004).

41 Kall, L., Krogh, A. & Sonnhammer, E. L. Advantages of combined transmembrane topology and signal peptide prediction--the Phobius web server. *Nucleic acids research* **35**, W429-432, doi:10.1093/nar/gkm256 (2007).

42 Bendtsen, J. D., Jensen, L. J., Blom, N., Von Heijne, G. & Brunak, S. Feature-based prediction of non-classical and leaderless protein secretion. *Protein Eng. Des. Sel.* **17**, 349-356, doi:10.1093/protein/gzh037 (2004).

43 Rawlings, N. D., Waller, M., Barrett, A. J. & Bateman, A. MEROPS: the database of proteolytic enzymes, their substrates and inhibitors. *Nucleic Acids Res.* **42**, 23 (2014).

44 Wylie, T. *et al.* NemaPath: online exploration of KEGG-based metabolic pathways for nematodes. *BMC genomics* **9**, 525, doi:10.1186/1471-2164-9-525 (2008).

45 Kanehisa, M., Goto, S., Sato, Y., Furumichi, M. & Tanabe, M. KEGG for integration and interpretation of large-scale molecular data sets. *Nucleic acids research* **40**, D109-114, doi:10.1093/nar/gkr988 (2012).

46 Ashburner, M. *et al.* Gene ontology: tool for the unification of biology. The Gene Ontology Consortium. *Nature genetics* **25**, 25-29, doi:10.1038/75556 (2000).

47 Hunter, S. *et al.* InterPro in 2011: new developments in the family and domain prediction database. *Nucleic acids research* **40**, D306-312, doi:10.1093/nar/gkr948 (2012).

48 Quevillon, E. *et al.* InterProScan: protein domains identifier. *Nucleic acids research* **33**, W116-120, doi:10.1093/nar/gki442 (2005).

49 Prufer, K. *et al.* FUNC: a package for detecting significant associations between gene sets and ontological annotations. *BMC bioinformatics* **8**, 41, doi:10.1186/1471-2105-8-41 (2007).

50 Dalzell, J. J. *et al.* RNAi effector diversity in nematodes. *PLoS neglected tropical diseases* **5**, e1176, doi:10.1371/journal.pntd.0001176 (2011).

51 Martin, D. M., Miranda-Saavedra, D. & Barton, G. J. Kinomer v. 1.0: a database of systematically classified eukaryotic protein kinases. *Nucleic acids research* **37**, D244-250, doi:10.1093/nar/gkn834 (2009).

52 Miranda-Saavedra, D. & Barton, G. J. Classification and functional annotation of eukaryotic protein kinases. *Proteins* **68**, 893-914, doi:10.1002/prot.21444 (2007).

53 Eddy, S. R. Accelerated Profile HMM Searches. *PLoS computational biology* **7**, e1002195, doi:10.1371/journal.pcbi.1002195 (2011).

54 Moriya, Y., Itoh, M., Okuda, S., Yoshizawa, A. C. & Kanehisa, M. KAAS: an automatic genome annotation and pathway reconstruction server. *Nucleic acids research* **35**, W182-185, doi:10.1093/nar/gkm321 (2007).

55 Tyagi, R., Rosa, B. A., Lewis, W. G. & Mitreva, M. Pan-phylum Comparison of Nematode Metabolic Potential. *PLoS neglected tropical diseases* **9**, e0003788, doi:10.1371/journal.pntd.0003788 (2015).

56 Yeh, I., Hanekamp, T., Tsoka, S., Karp, P. D. & Altman, R. B. Computational analysis of *Plasmodium falciparum* metabolism: organizing genomic information to facilitate drug discovery. *Genome research* **14**, 917-924, doi:10.1101/gr.2050304 (2004).

57 Taylor, C. M. *et al.* Discovery of anthelmintic drug targets and drugs using chokepoints in nematode metabolic pathways. *PLoS pathogens* **9**, e1003505, doi:10.1371/journal.ppat.1003505 (2013).

58 Berman, H. M. *et al.* The Protein Data Bank. *Nucleic acids research* **28**, 235-242 (2000).

59 Law, V. *et al.* DrugBank 4.0: shedding new light on drug metabolism. *Nucleic acids research* **42**, D1091-1097, doi:10.1093/nar/gkt1068 (2014).

60 Lopez, R., Silventoinen, V., Robinson, S., Kibria, A. & Gish, W. WU-Blast2 server at the European Bioinformatics Institute. *Nucleic acids research* **31**, 3795-3798 (2003).

61 Koutsovoulos, G., Makepeace, B., Tanya, V. N. & Blaxter, M. Palaeosymbiosis revealed by genomic fossils of *Wolbachia* in a strongyloidean nematode. *PLoS genetics* **10**, e1004397, doi:10.1371/journal.pgen.1004397 (2014).

62 Dodt, M., Roehr, J. T., Ahmed, R. & Dieterich, C. Flexbar − flexible barcode and adapter processing for next-generation sequencing platforms. *MDPI Biology* **1**, 895-905 (2012).

63 Li, H. & Durbin, R. Fast and accurate short read alignment with Burrows-Wheeler transform. *Bioinformatics* **25**, 1754-1760, doi:10.1093/bioinformatics/btp324 (2009).

64 McKenna, A. *et al.* The Genome Analysis Toolkit: a MapReduce framework for analyzing next-generation DNA sequencing data. *Genome research* **20**, 1297-1303, doi:10.1101/gr.107524.110 (2010).

65 Cingolani, P. *et al.* A program for annotating and predicting the effects of single nucleotide polymorphisms, SnpEff: SNPs in the genome of *Drosophila melanogaster* strain w1118; iso-2; iso-3. *Fly* **6**, 80-92, doi:10.4161/fly.19695 (2012).

66 Purcell, S. *et al.* PLINK: a tool set for whole-genome association and population-based linkage analyses. *Am J Hum Genet* **81**, 559-575 (2007).

67 Danecek, P. *et al.* The variant call format and VCFtools. *Bioinformatics* **27**, 2156-2158, doi:10.1093/bioinformatics/btr330 (2011).

68 Bandelt, H. J., Forster, P. & Rohl, A. Median-joining networks for inferring intraspecific phylogenies. *Mol Biol Evol* **16**, 37-48 (1999).

69 Wang, D., Zhang, Y., Zhang, Z., Zhu, J. & Yu, J. KaKs_Calculator 2.0: a toolkit incorporating gamma-series methods and sliding window strategies. *Genomics Proteomics Bioinformatics* **8**, 77-80, doi:10.1016/S1672-0229(10)60008-3 (2010).

70 Tajima, F. Statistical method for testing the neutral mutation hypothesis by DNA polymorphism. *Genetics* **123**, 585-595 (1989).

71 RefCov v. 0.3 (http://gmt.genome.wustl.edu/gmt-refcov).

72 Anders, S., Pyl, T. P. & Huber, W. HTSeq — A Python framework to work with high-throughput sequencing data. *bioRxiv*, doi:10.1101/002824. (2014).

73 Anders, S. & Huber, W. Differential expression analysis for sequence count data. *Genome biology* **11**, R106, doi:10.1186/gb-2010-11-10-r106 (2010).

74 Anders, S. *et al.* Count-based differential expression analysis of RNA sequencing data using R and Bioconductor. *Nature protocols* **8**, 1765-1786, doi:10.1038/nprot.2013.099 (2013).
